# 2-DE-based proteomic analysis of protein changes associated with etiolated mesocotyl growth in *Zea mays*

**DOI:** 10.1186/s12864-019-6109-z

**Published:** 2019-10-22

**Authors:** Liangjie Niu, Zhaokun Wu, Hui Liu, Xiaolin Wu, Wei Wang

**Affiliations:** State Key Laboratory of Wheat and Maize Crop Science, College of Life Sciences, Henan Agricultural University, Zhengzhou, 450002 China

**Keywords:** Mesocotyl growth, 2-DE, Proteomic analysis, Differential abundance protein (DAP), *Zea mays*

## Abstract

**Background:**

The mesocotyl connects the coleoptilar node and the basal part of the seminal root of maize (*Zea mays*) seedling. The mesocotyl pushes the shoot of the seedling out of the soil during seed germination; thus, its growth is highly related to deep-sowing tolerance. Although many studies on the maize mesocotyl have been carried out at physiological and molecular levels, the proteomic changes associated with cellular and physiological activities during mesocotyl growth are still unknown.

**Results:**

In the present study, the maize hybrid Zhengdan 958 was used to study mesocotyl growth and accompanying protein changes*.* The dark-grown etiolated mesocotyls exhibited a slow-fast-slow feature, with significant changes in the levels of indole-3-acetic acid (IAA) and cellulose and the activity of peroxidase (POD). In particular, POD activity increased with mesocotyl growth, showing higher activity at the mature (lower) end of the mesocotyl. For the proteomic analysis, soluble proteins were extracted from etiolated mesocotyls dark-grown for 48 h, 84 h, and 132 h, corresponding to the initial, rapid, and slow growth periods, respectively, and subjected to separation by two-dimensional gel electrophoresis (2-DE). As a result, 88 differentially abundant proteins (DAPs) were identified using MALDI-TOF-TOF analysis. At 48 h, most DAPs were stress proteins, heat shock proteins and storage proteins; at 84 h, oxidation/reduction proteins, carbohydrate biogenesis-related proteins and cytoskeleton-related proteins were highly accumulated; at 132 h, the most striking DAPs were those involved in the synthesis and modification of the cell wall and the biogenesis of carbohydrates. Gene ontology (GO) analysis showed that changes in the abundance and proportion of DAPs were consistent with cellular and physiological activities and biological processes during mesocotyl growth. The accumulation of nine DAPs of interest was verified by immunoblotting and RT-qPCR.

**Conclusions:**

The present study revealed that the protein patterns in 2-D gels differed greatly with mesocotyl growth. At different growth periods, a specific set of DAPs participate in various biological processes and underlie the cellular and physiological activities of the mesocotyl. These results contributed to the understanding of mesocotyl growth and the cultivation of maize lines with deep-sowing tolerance.

## Background

The maize mesocotyl is an organ that connects the coleoptilar node and the basal part of the seminal root in young seedlings [1]. The mesocotyl consists of one centrally localized vascular stele and a hollow cylinder of cortical cells bounded by an epidermal layer [2]. The mesocotyl can be longitudinally divided into three parts: the apical meristematic part, the middle elongation part and the lower mature part [3]. This structure plays two important roles during early seedling establishment. First, the elongating mesocotyl elevates the coleoptile and enclosed plumule towards the soil surface [4]; and second, the cortical aerenchyma inside the mesocotyl supports the initiation and growth of adventitious roots [5]. Therefore, the elongated mesocotyl is highly related to maize deep-sowing tolerance.

Maize mesocotyl growth is attributable to cell division and turgor-driven wall expansion, which is regulated by internal and environmental factors, such as phytohormones [6–9], light [10–13] and gravity [14–16]. In particular, its growth is inhibited by red light via phytochrome action [17] and along with the decrease in the level of auxins in epidermal cells [12, 18, 19]. Moreover, maize mesocotyl growth is regulated by various phytohormones [10, 12, 20], auxin-binding proteins [21, 22] and vacuolar H^+^-ATPase [11]. In particular, polyamine oxidases in mesocotyl epidermal cells are actively involved in cell wall stiffening processes during the light-stimulated inhibition of mesocotyl growth [23]. Additionally, several quantitative trait loci or genes related to deep-sowing tolerance have been identified [24, 25]. Although many studies on maize mesocotyl growth have been carried out at physiological and molecular levels, there remains a lack of proteomic analysis of protein changes associated with cellular and physiological activities in the mesocotyl.

In the present study, the dark-grown (etiolated) mesocotyls of maize were used for two-dimensional electrophoresis (2-DE)-based proteomic analysis to identify differentially abundant proteins (DAPs) during mesocotyl growth. The DAPs were functionally classified with gene ontology (GO) analysis. Our results revealed that unique sets of DAPs played crucial roles during different growth periods of the mesocotyl.

## Results

### Maize mesocotyl growth changes with sowing depth

**Fig. 1:**
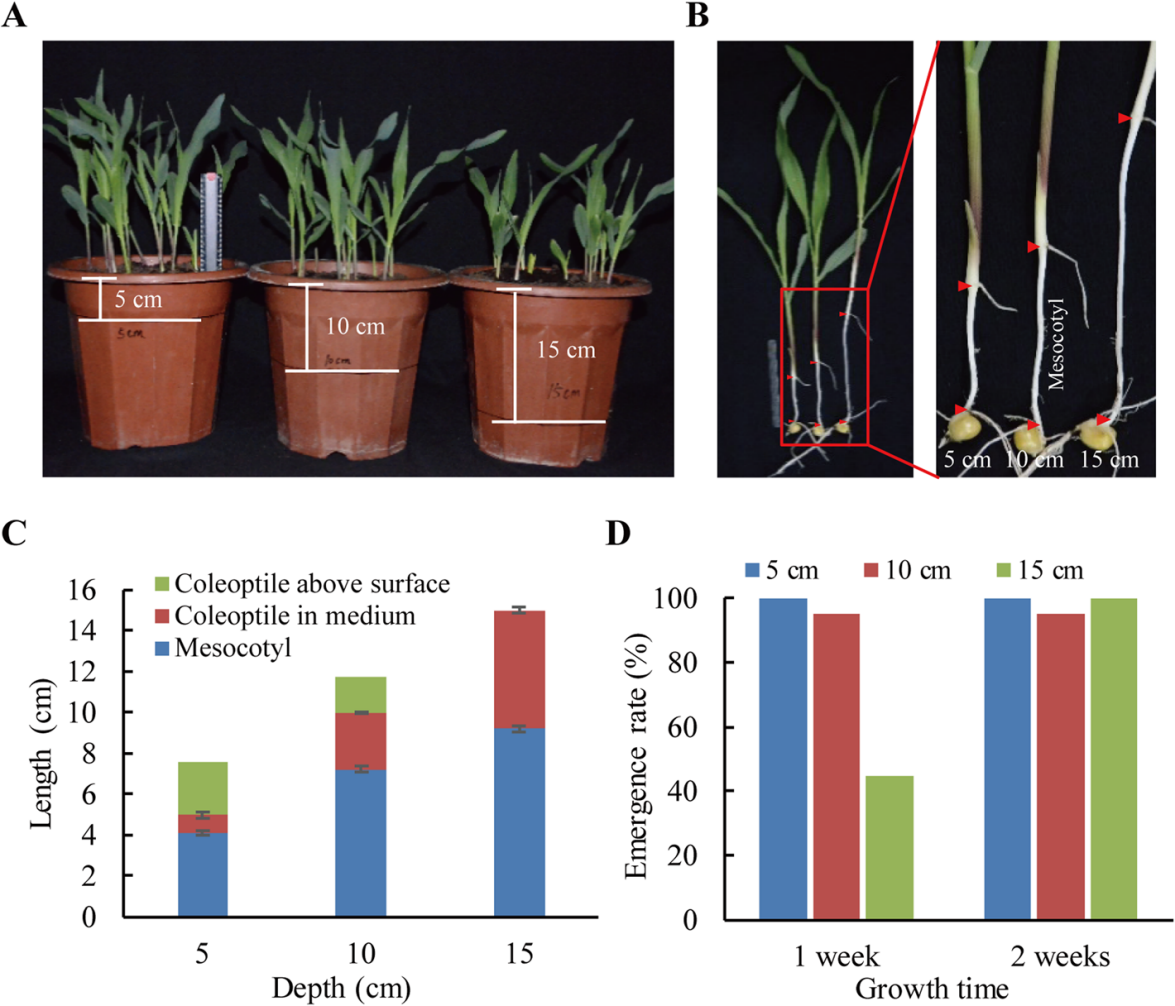
Mesocotyl length at different sowing depths. Maize seedlings were grown under a photoperiod with 16 h light (80–100 μE m^− 2^ s^− 1^, 28 °C). After two weeks, the lengths of mesocotyl and coleoptile and the germination rate were measured. **a** Seedlings at different sowing depths. **b** Three representative seedlings showing changes in mesocotyl length with sowing depth. **c** The lengths of mesocotyl and coleoptile at different sowing depths. **d** Germination rate at different sowing depths. The data represented the results from three independent experiments (mean ± SE), and at least 15 seedlings were measured in each assay

To determine the contribution of mesocotyl elongation to seed germination, we compared the length changes of the mesocotyl and the coleoptile at 5, 10 and 15 cm sowing depths (Fig. 1a,b). The assay was performed at 14 days after sowing when the lengths of mesocotyl and coleoptile were fixed. The length of the mesocotyl and the coleoptile increased with sowing depth, especially the mesocotyl. In all cases, the mesocotyl made a greater contributed to seed germination in soil than did the coleoptile (Fig. 1c) because the mesocotyl consistently grew in the soil (dark), whereas the coleoptile growth was inhibited by light upon emergence. Seedling emergence was significantly delayed at a 15-cm depth, resulting in poorly uniform seedlings; however, seedling emergence at 14 days showed no significant difference among different sowing depths (Fig. 1d).

### Maize mesocotyl growth in vivo and in vitro

**Fig. 2:**
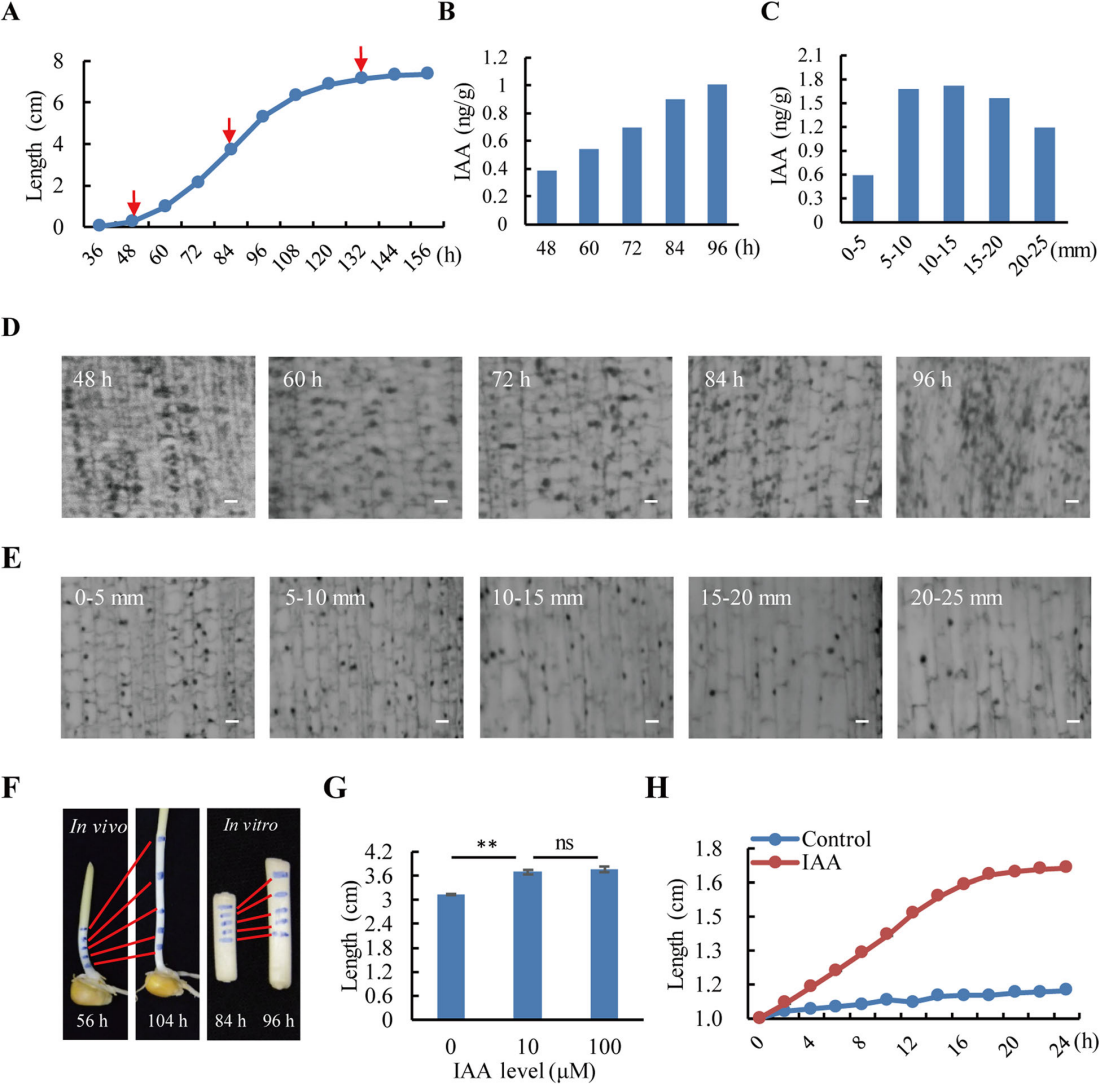
Growth analysis of the etiolated mesocotyl. After imbibition for 12 h, the maize seeds were germinated in darkness at 25 °C for a week. **a** Growth curve of the mesocotyl in vivo. The arrows indicate the sampling time (48 h, 84 h and 132 h) for proteomic analysis. **b** IAA content change with mesocotyl growth. IAA was determined per fresh weight. **c** IAA content in different parts of mesocotyl. The mesocotyls were sampled from 84-h-old etiolated seedlings. IAA was determined per fresh weight. **d** Light microscopy of the mesocotyl, showing the changes in cell morphology at the apical 5 mm part with mesocotyl growth. Bar = 10 μm. **e** Light microscopy of the mesocotyls, showing the changes in cell morphology at different parts of the 84-h-old mesocotyls. Bar = 10 μm. **f** Mesocotyl growth in vivo for 48 h and in vitro for 12 h. The mesocotyls were marked to show the differential elongation of different parts. **g** IAA effect on mesocotyls grown in vitro for 12 h. ** denotes *p* < 0.01, ns stands for not significant. **h** Growth curve of the mesocotyl in vitro with or without 10 μM IAA. The mesocotyl segments were sampled from 84-h-old etiolated seedlings

The etiolated mesocotyl showed a slow-fast-slow growth pattern during the initial growth period (36–48 h), the rapid growth period (48–132 h) and the slow growth period (132–156 h). The final length was 7.40 ± 0.15 cm, with a maximum growth rate at 84 h (1.30 ± 0.16 mm/h) (Fig. 2a). The fresh and dry weights and cellulose content of the mesocotyl significantly increased with growth, but the protein content and the relative water content (RWC) changed little from 84 h onward (Table 1a).

**Table 1:** Analysis of maize mesocotyl growth

A. Mesocotyl in vivo					
Growth time	Fresh weight (mg)	Dry weight (mg)	RWC (%)	Protein mg/g dry weight	Cellulose (%) (per dry weight)
48 h	27.78 ± 2.41^a^	2.79 ± 0.19 ^a^	90.86 ± 0.33 ^a^	90.22 ± 8.28 ^a^	31.04 ± 3.41 ^a^
84 h	115.93 ± 2.10 ^b^	8.65 ± 0.59 ^b^	93.06 ± 0.36 ^b^	41.14 ± 11.70 ^b^	40.65 ± 0.79 ^b^
132 h	269.08 ± 9.33 c	22.24 ± 0.88 ^c^	92.36 ± 0.38 ^b^	38.48 ± 4.30 ^b^	24.59 ± 6.38 ^c^
B. Mesocotyl in vitro					
Incubation time	Fresh weight (mg)	Dry weight (mg)	RWC (%)	Protein mg/g dry weight	Cellulose (%) (per dry weight)
0 h	32.78 ± 0.52 ^a^	2.35 ± 0.04 ^a^	92.84 ± 0.11 ^a^	0.24 ± 0.03 ^a^	No detection
12 h, − IAA	38.23 ± 0.50 ^b^	2.47 ± 0.08 ^a^	93.53 ± 0.18 ^b^	0.16 ± 0.04 ^a^	23.93 ± 1.52 ^a^
12 h, + IAA	52.19 ± 3.81 ^c^	2.73 ± 0.02 ^a^	94.75 ± 0.39 ^c^	0.15 ± 0.02 ^a^	19.50 ± 2.95 ^b^

Assays were repeated at least three times. Values having different superscripts are significantly different at *p* < 0.01

The IAA content in elongating mesocotyls was analyzed per fresh weight. As expected, IAA increased rapidly during 48–96 h, corresponding to the rapid growth period (Fig. 2b). In 84-h-old mesocotyls, the IAA content varied in different parts, with the highest in the 5–20 mm region of the elongation zone (Fig. 2c). Light microscopy showed that the size of mesocotyl cells increased with time (Fig. 2d) and with cell maturation (from upper to bottom) (Fig. 2e). Thus, the size of mesocotyl cells was positively correlated with the IAA content. Additionally, the upper half of the mesocotyl exhibited quick division and growth both in vivo and in vitro (Fig. 2f).

Mesocotyl segments displayed rapid growth in the presence of IAA, and there was no significant difference between 10 μM and 100 μM (Fig. 2g). Therefore, 10 μM IAA was used in further analysis. Similar to in vivo growth, mesocotyl in vitro also showed a slow-fast-slow growth pattern. After incubation for 24 h, the length of mesocotyl segments increased from 10 mm to 16.66 ± 0.79 mm, with the maximum growth rate at 12 h (0.24 ± 0.01 mm/h) (Fig. 2h). The fresh weight, RWC and cellulose content of the mesocotyl segments significantly increased with in vivo growth, but the protein content decreased (Table 1b).

### Protein changes during maize mesocotyl growth

**Fig. 3:**
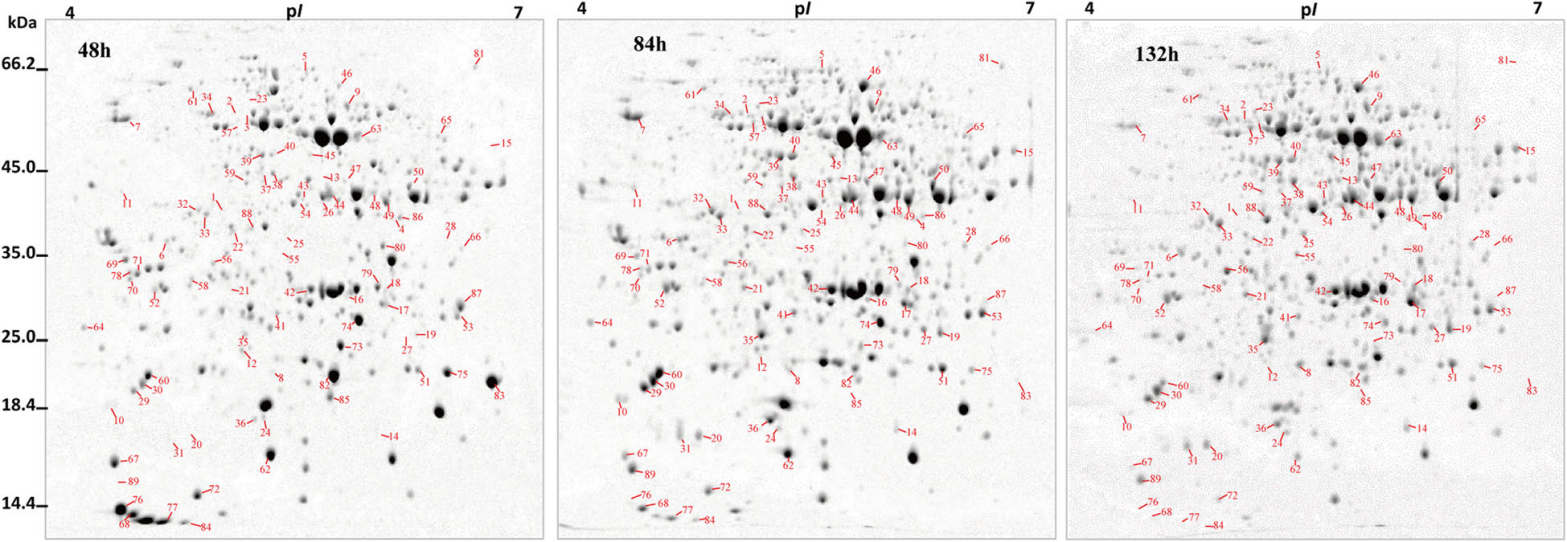
The 2-DE analysis of mesocotyl growth. Mesocotyls were sampled from 48 h-, 84 h- and 132 h-old etiolated maize seedlings. Representative gels from three independent experiments are shown. The proteins (600 μg) were resolved by IEF using 11 cm pH 4–7 IPG strips. SDS-PAGE was carried out on 12.5% resolving gels. The proteins were visualized using CBB R350. Differentially abundant spots with at least 2-fold abundance changes are highlighted by numbers

**Fig. 4:**
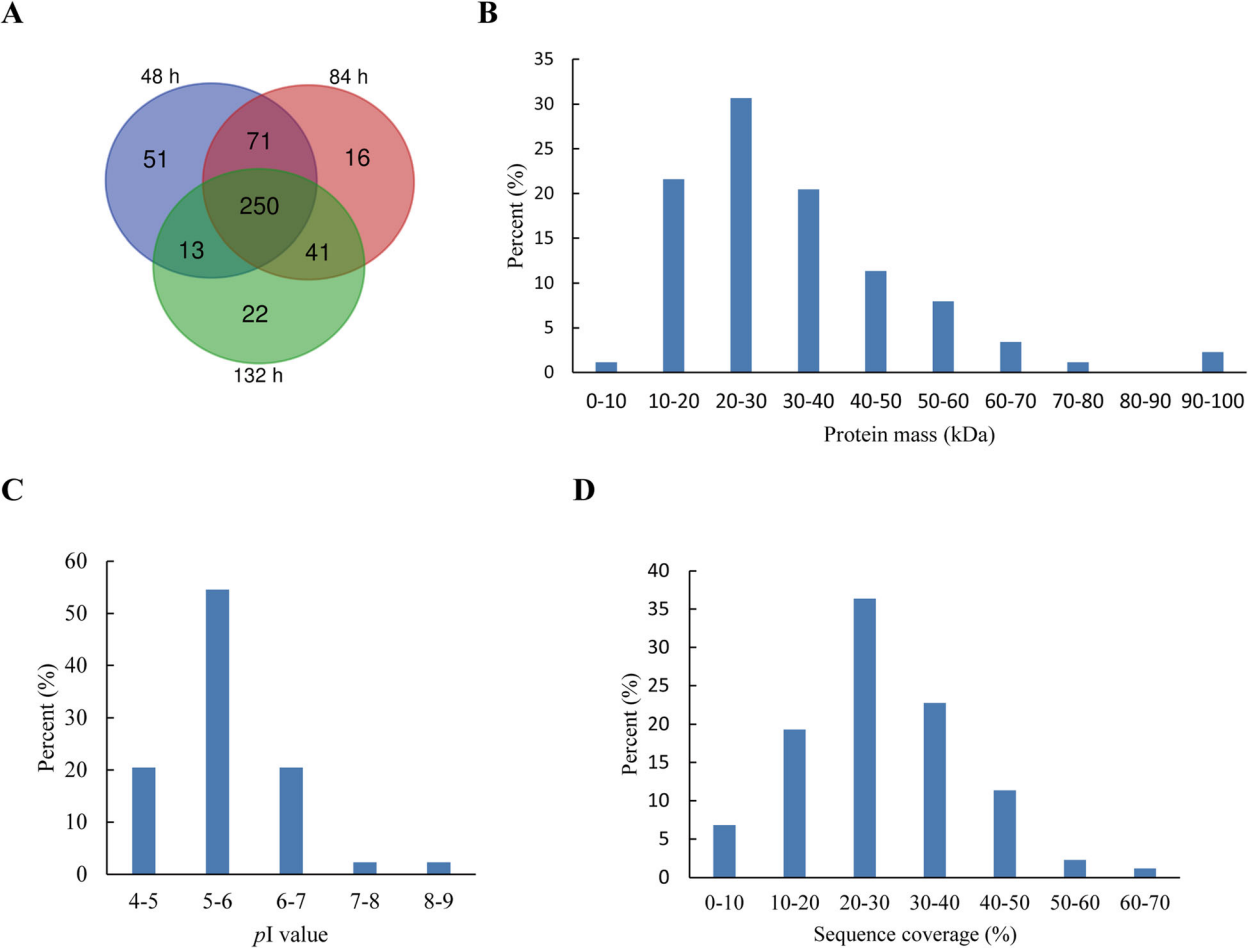
Summary of DAPs identified in mesocotyls. **a** Venn diagrams of proteins resolved in 2-DE gels of the mesocotyls sampled at three time points. **b** MW distribution of DAPs. **c** p*I* distribution of DAPs. **d** coverage (%) of the matched sequences of DAPs

The protein accumulation differences of the mesocotyls at 48 h, 84 h and 132 h were analyzed by 2-DE with pH 4–7 IPG strips. In general, the distribution patterns of the protein spots on 2-DE gels were similar among the three growth periods (Fig. 3, Additional file 4: Figure S1), but each period had a specific set of differential abundance proteins (DAPs) (Fig. 4a).

After ANOVA and Student’s t test (*p* < 0.05; *n* = 3), 89 DAPs with at least 2-fold differences in relative abundance (Additional file 1: Table S1) were selected for protein identification with MALDI-TOF/TOF analysis. Except for spot 64, 88 other DAPs were successfully identified (Additional file 2: Table S2). These DAPs exhibited good coverage regarding molecular weight (MW) and isoelectric point (p*I*) (Fig. 4b,c). Most DAPs had a high sequence coverage (20%) with at least three matched sequences (Fig. 4d). In addition, there was high reproducibility among the three independent biological replicates on 2-DE, i.e., 92.4, 76.3, and 76.7% for the 48 h, 84 h and 132 h gels, respectively (Additional file 1: Table S1).

**Fig. 5:**
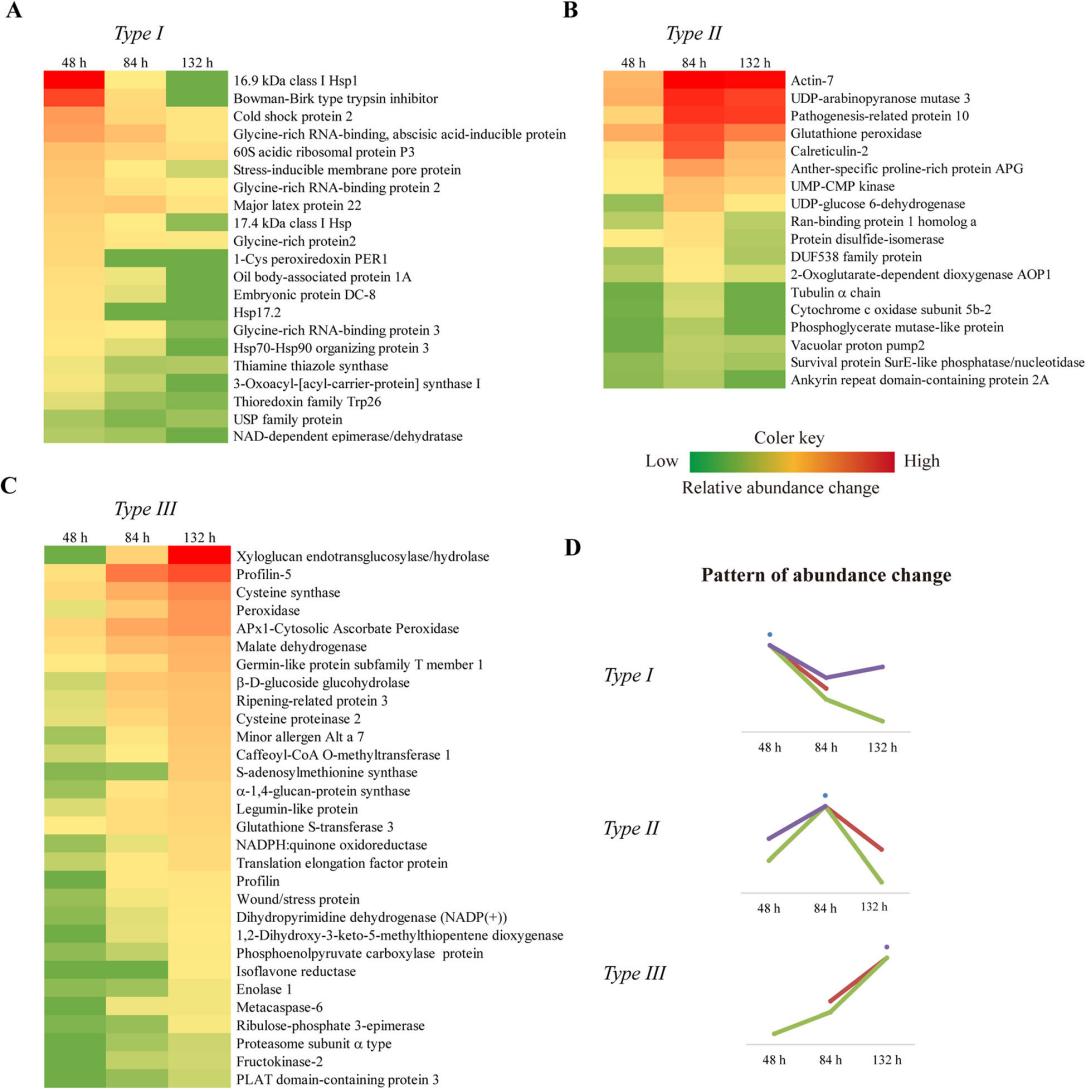
Relative abundances of the DAPs during mesocotyl growth. The heatmaps are presented in matrix format, in which the colors represent the abundance level of each protein. **a** type IDAPs. **b** type II DAPs. **c** type III DAPs. **d** pattern of abundance change

According to the changes in the patterns of protein abundance, the 88 DAPs were classified into three types: Ι, proteins with gradually decreased abundance or only observed at 48 h; ΙΙ, proteins with initially increased and gradually declined abundance or only observed at 84 h; ΙΙΙ, proteins with gradually increased abundance or only observed at 132 h (Fig. 5; Table 2).

**Table 2:** Summary of the DAPs associated to mesocotyl growth in maize

Spot	UniProtKB accession	Protein	Function or biological process involved
*Type Ι DAPs*			
12	K7V2N6	USP family protein	Response to stress
62	P10979	Glycine-rich RNA-binding, ABA-inducible protein	
73	B4F976	17.4 kDa class I HSP	
74	C0PLI2	Cold shock protein 2	
75	A0A1D6MRR5	Stress-inducible membrane pore protein	
81	A0A1D6HEP0	Hsp70-Hsp90 organizing protein 3	
82,83	B6SIX0	16.9 kDa class I HSP 1	
85	A0A1D6MLV9	HSP 17.2	
58	B4FPP1	Thioredoxin family Trp26	Cell redox homeostasis, defense response
59	B4G015	Thiamine thiazole synthase	Thiamine biosynthesis
60	Q19VG6	Major latex protein 22	abscisic acid-activated signaling pathway, defense response
65	K7V9P7	3-Oxoacyl-[acyl-carrier-protein] synthase I chloroplastic	Fatty acid biosynthesis
66	A0A1D6L9Y9	NAD-dependent epimerase/dehydratase	Colanic acid and lipopolysaccharide biosynthesis
67,89	A0A1D6LNJ7 B4G1B3	60S acidic ribosomal protein P3	Structural constituent of ribosome
68,76,77,84	A0A1D6MM79	Bowman-Birk type trypsin inhibitor	Serine-type endopeptidase inhibitor activity
69,71	A0A1D6IV87	Glycine-rich protein2	Mitochondrial mRNA modification
72	B6SIF0	Glycine-rich RNA-binding protein 2	
70,78	A0A1D6MXZ7	Glycine-rich RNA-binding protein 3	
79	B4FFZ9	Oil body-associated protein 1A	Storage protein
80,86	B6SGN7, A0A1D6JQ00	Embryonic protein DC-8	
87	A2SZW8	1-Cys peroxiredoxin PER1	Cell redox homeostasis
*Type II DAPs*			
2,3	A0A1D6GIG4 C0PKT5	Tubulin α chain	Structural constituent of cytoskeleton
39,40	A0A1D6FW13, A0A1D6EHT1	Actin-7	
4	A0A1D6EG37	Phosphoglycerate mutase	Carbohydrates metabolism
5,23	A0A1D6P248	Vacuolar proton pump2	ATP metabolic process
6	A0A1D6JZE5	Ran-binding protein 1	Intracellular transport, positive regulation of GTPase activity
7	A0A1D6EN35	Calreticulin-2	Protein folding
8	B4FMB1	DUF538 family protein	Uncharacterized
9	B7ZYX8	UDP-glucose 6-dehydrogenase	Glycosaminoglycan biosynthesis
10	K7 V763	Cytochrome c oxidase subunit 5b-2	Mitochondrial electron transport
11	B4G137	Ankyrin repeat domain-containing protein 2A	Protein targeting to chloroplast
24,36	Q29SB6	Pathogenesis-related protein 10	Response to stress
32	B4F9E3	Anther-specific proline-rich protein APG	Hydrolase activity, acting on ester bonds
34	A0A1D6G329	Survival protein SurE-like phosphatase/nucleotidase	Hydrolase activity
37	B4FN73	2-Oxoglutarate-dependent dioxygenase AOP1	Auxin catabolic process
41	A0A1D6QDC6	UMP-CMP kinase	Nucleotide biosynthesis
50	B4G039	UDP-arabinopyranose mutase 3	UDP-L-arabinose metabolic process, plant-type cell wall biogenesis
51	Q6JAH6	Glutathione peroxidase	Cell redox homeostasis
61	A0A1D6F5C2	Protein disulfide-isomerase	
*Type III DAPs*			
1,15,33	A0A1D6E530, B6THU9	Peroxidase	Response to stress
14	C4J9R0	PLAT domain-containing protein 3	
31	K7U5W7	Wound/stress protein	
42	B6TM55	APx1-cytosolic ascorbate peroxidase	
13,47	B4FQX1	α-1,4-glucan-protein synthase	UDP-L-arabinose metabolic process, plant-type cell wall biogenesis
16	A0A1D6I841	Ribulose-phosphate 3-epimerase	Carbohydrate metabolic process
49	A0A1R3MB28	Malate dehydrogenase	
46	P49235	β-D-glucoside glucohydrolase	
29,30	Q9FR39	Profilin-5	
17	B4FRS8	Germin-like protein subfamily T member 1	Manganese ion binding, nutrient reservoir activity
18	A0A1D6N0U0	NADPH:quinone oxidoreductase	Oxidation-reduction process
19	B4FWD0	Minor allergen Alt a 7	
20	A0A1D6GCC8	Profilin	Regulation of actin cytoskeleton organization
21	A0A1D6JPD5	1,2-Dihydroxy-3-keto-5-methylthiopentene dioxygenase	Methionine metabolic process
22,28,88	B4FTH5, B4FHS5, B6T2W7	Xyloglucan endotransglucosylase/hydrolase	Cell wall biogenesis, xyloglucan metabolic process
25	B6TAJ3	Proteasome subunit α type	Proteasomal protein catabolic process
26	A0A1D6LDR6	Fructokinase-2	Carbohydrate metabolic process
27	A0A1D6N9H5	Metacaspase-6	Cysteine-type peptidase activity
35	A0A1D6GNR3	Ripening-related protein 3	Defense response to fungus
38	A0A1D6GCC4	Translation elongation factor family protein	Translation elongation factor activity
43	A0A1D6ECS1	Phosphoenolpyruvate carboxylase family protein	Carbon fixation in CAM and C4 organisms
44	B8A377	Cysteine synthase	Cysteine biosynthetic
45	B4FZU9	Dihydropyrimidine dehydrogenase (NADP(+))	Cellular response to nitrogen levels, thymine catabolic process, uracil catabolic process
48	Q84TL7	Legumin-like protein	nutrient reservoir activity
52	A0A1D6I5B2	Cysteine proteinase 2	Cysteine-type peptidase activity
53	B4FK84	Glutathione S-transferase 3	Glutathione metabolic process
54	B4FD74	Isoflavone reductase-like1	2′-hydroxyisoflavone reductase activity, defense response
56	B6UF45	Caffeoyl-CoA O-methyltransferase 1	Feruloylated polysaccharides synthesis
57	A0A1D6NVW3	Enolase 1	Glycolytic process
63	B4FIE9	S-adenosylmethionine synthase	S-adenosylmethionine biosynthetic process, one-carbon metabolic process

Type Ι DAPs included heat shock proteins, stress proteins, storage proteins, protein biogenesis/degradation-related proteins, oxidation-reduction proteins and carbohydrate biogenesis-related proteins (Additional file 5: Figure S2a). Type Ι DAPs were obviously crucial to the synthesis of proteins and building blocks and the energy metabolism required for cell division and expansion during mesocotyl growth. For example, the 16.9 kDa class I heat shock protein 1 (spots 82, 83) and the Bowman-Birk type trypsin inhibitor (spots 68, 76, 77, 84) exhibited approximately 57- and 40-fold changes in abundance, respectively.

Type ΙΙ DAPs mainly included oxidation-reduction proteins, increased carbohydrate biogenesis-related proteins and many cytoskeleton-related proteins (Additional file 5: Figure S2a). That is the biogenesis of the cell wall and cytoskeleton, and more energy continued to be necessary for the rapid growth of the mesocotyl. For example, actin (spots 39, 40), tubulin (spots 2, 3), calreticulin-2 (spot 7) and UDP-arabinopyranose mutase 3 (spot 50) showed increased abundance during the rapid growth period, and these proteins were all involved in cytoskeleton and cell wall biogenesis. Both V-ATPase subunits A and B (spots 5, 23), involved in cell wall loosening, and cytochrome c oxidase subunit 5b-2 (Spot 10), involved in mitochondrial electron transport, showed increased abundance during this period. Moreover, spot 37 was identified as the putative 2-oxoglutarate-dependent dioxygenase AOP1, which shows indole-3-acetaldehyde oxidase activity in the cytoplasm. This protein accumulated in high abundance during the rapid growth period according to the change in IAA level.

Type ΙΙΙ DAPs were mainly involved in protein biogenesis and degradation, cell wall biogenesis, carbohydrate biogenesis, cytoskeleton biogenesis and oxidation-reduction (Additional file 5: Figure S2a). Profilin (spot 20) and profilin-5 (spots 29, 30) can bind to actin and affect the structure of the cytoskeleton. In particular, the abundance of cell wall-related proteins and carbohydrate biogenesis-related proteins greatly changed. Xyloglucan endotransglucosylase/hydrolase (spots 22, 28, 88) and α-1,4-glucan-protein synthase (spots 13, 47) are involved in cell wall biogenesis, and peroxidase (spots 1, 15, 34, 43, 47) participated in decreased cell wall extensibility, and these proteins showed increased abundance during the slow growth period. Ribulose-phosphate 3-epimerase (spot 16), β-D-glucoside glucohydrolase (spot 46) and malate dehydrogenase (spot 49) are involved in cellular carbohydrate metabolic processes, and these activities are highly associated with organ growth and cell wall biogenesis, which is the most striking process during maize mesocotyl growth.

### Functional classification of DAPs with GO analysis

**Fig. 6:**
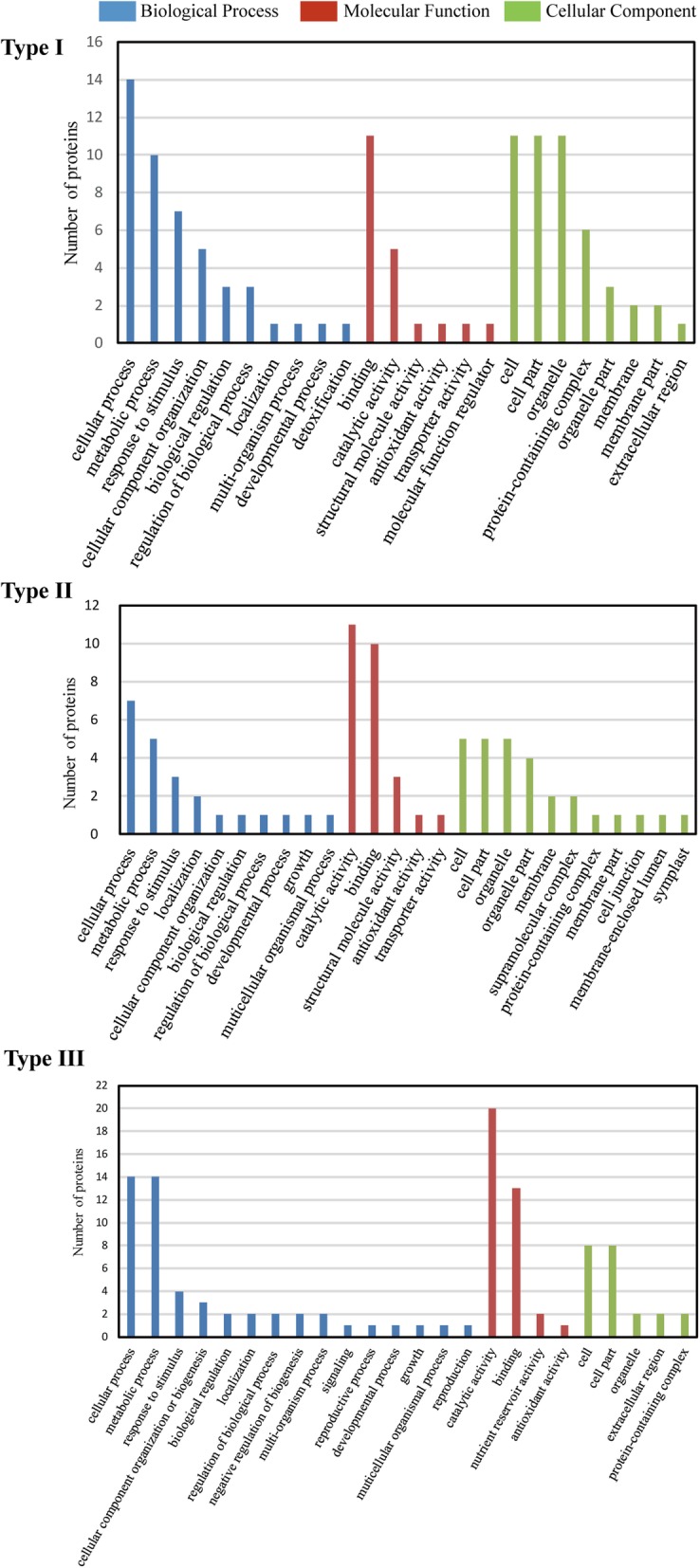
Functional categories of the DAPs during mesocotyl growth. The DAPs were classified by Blast2GO into biological processes, molecular functions and cellular components

The three types of DAPs were further classified using the Blast2GO software regarding biological process, molecular function and cellular component (Fig. 6). The top three common biological processes involving DAPs included cellular processes, metabolic processes and responses to stimuli. Diverse biological processes were initiated with mesocotyl growth, such as the negative regulation of biogenesis and signaling, which were exclusively associated with type III DAPs.

The major molecular functions of the DAPs included catalytic activity, binding and antioxidant activity, but each type of DAP involved specific functions (Additional file 5: Figure S2a). For example, type II and III DAPs shared structural molecule activities and transporter activities, whereas nutrient reservoir activity was specific to type III (storage proteins).

Regarding cellular components, the DAPs were most associated with the nucleus, microtubule, cytoplasm and cell wall, suggesting that these components were closely related to the growth of the maize mesocotyl. However, each DAP type had specific localizations, as shown by the subcellular location analysis (Additional file 5: Figure S2b).

### Verification of DAPs associated with mesocotyl growth

**Fig. 7:**
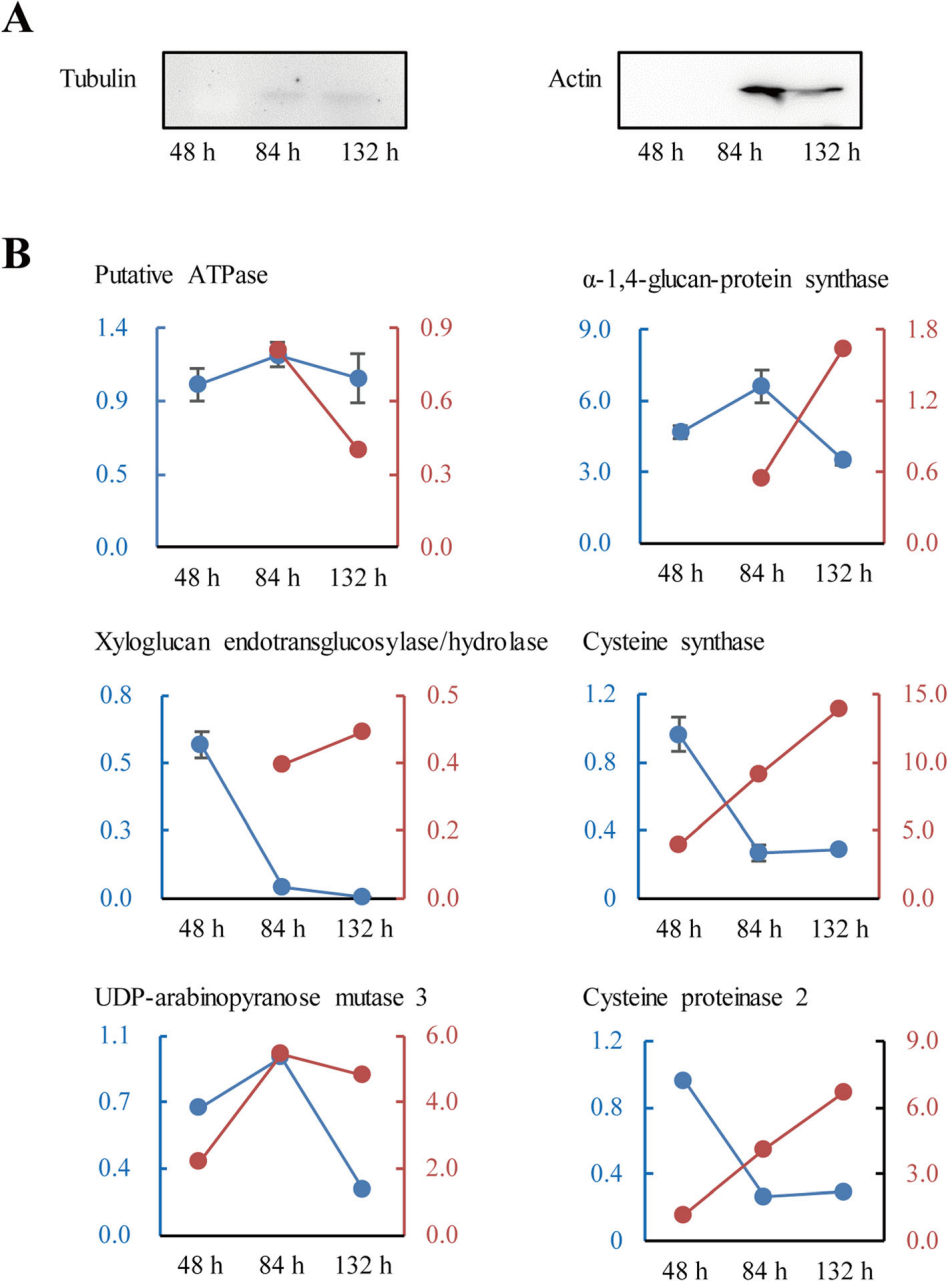
Verification of the selected DAPs during mesocotyl growth. **a** Immunoblot detection of actin and tubulin. **b** RT-qPCR of the mRNAs of six DAPs. Red and blue lines represent the levels of proteins and mRNAs, respectively

**Fig. 8:**
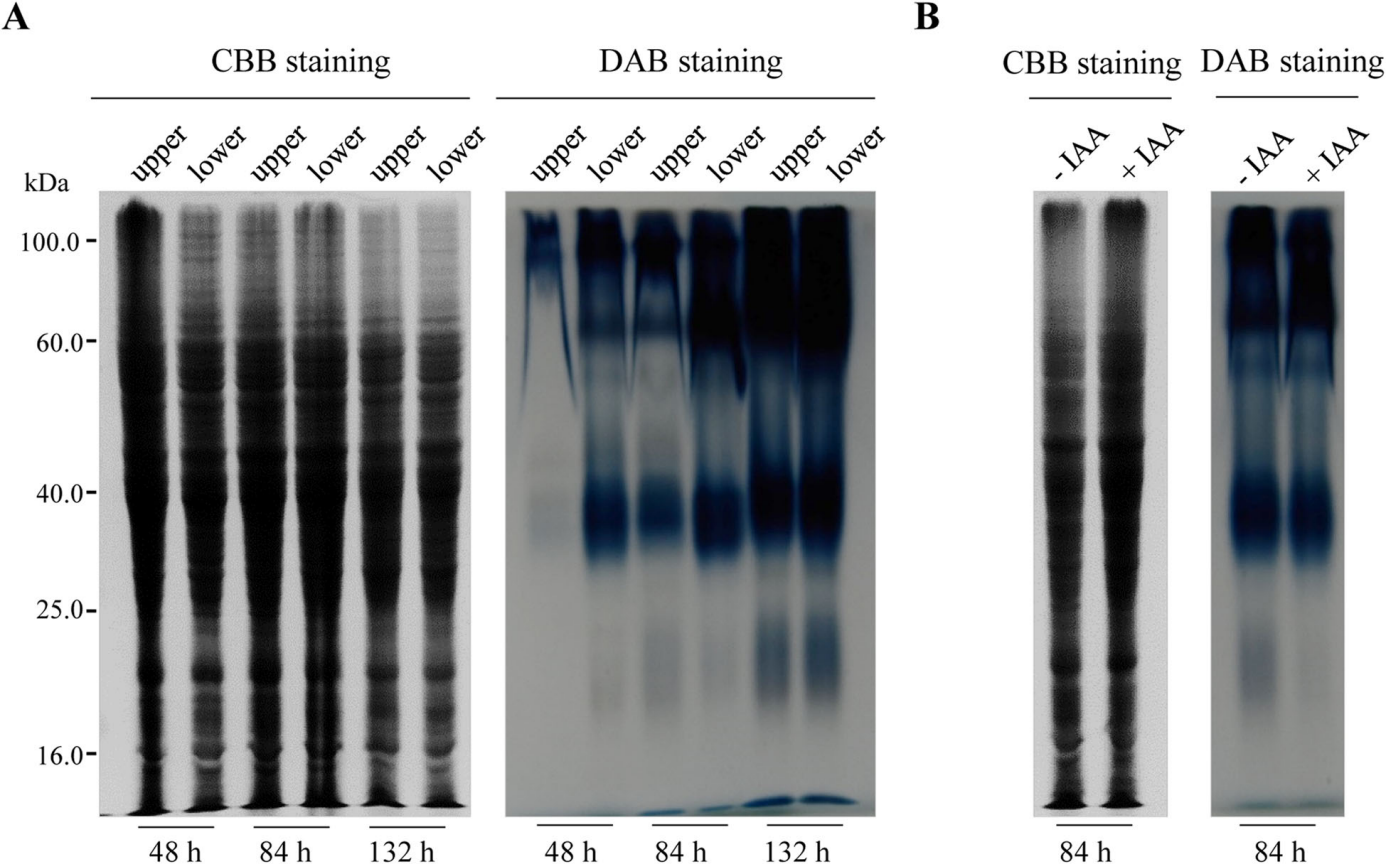
POD isozyme activity assay of maize mesocotyl. The mesocotyls were divided into the upper half and lower halves and used for SDS-PAGE and isozyme activity assays. **a** Mesocotyls sampled from 48 h-, 84 h- and 132-h-old etiolated seedlings. **b** Mesocotyl segments sampled from 84 h-old etiolated seedlings and incubated for 12 h with or without 10 μM IAA

Nine DAPs of interest were analyzed with immunoblotting, RT-qPCR or enzyme activity assays. Immunoblot analysis revealed that actin and tubulin were highly accumulated at 84 h and 132 h during mesocotyl growth (Fig. 7a), which was consistent with the proteomic results. RT-qPCR showed that the mRNA expression of six DAPs occurred obviously earlier than the accumulation of corresponding proteins, which partly supported the proteomic results (Fig. 7b). Finally, POD isozyme activity increased with mesocotyl growth (Fig. 8), which was consistent with the abundance changes in this protein revealed by 2-DE. Particularly, POD activity was higher at the lower part than at the upper part of the mesocotyl, which was in line with the fact that mesocotyl cells at the lower half matured earlier. Moreover, the POD isozyme activity of mesocotyl segments also increased in the presence of IAA.

## Discussion

The maize mesocotyl is highly related to maize deep-sowing tolerance [26–28]. This structure rapidly elongates during seed germination in the dark (in soil) and elevates the coleoptile (and the enclosed plumule) towards the soil surface. The elongating mesocotyl remains in soil throughout seed germination and seedling establishment. We showed here that mesocotyl growth was enhanced with depth (Fig. 1) by mimicking natural seed germination. Regardless of depth, the etiolated mesocotyl never penetrated the surface of the soil. Therefore, studying the growth of the etiolated mesocotyl has practical significance.

Numerous studies have shown that auxin promotes the growth of the maize mesocotyl. In the classical acid-growth theory, auxin activates the plasma-membrane H^+^-ATPase, resulting in membrane hyperpolarization, apoplastic acidification and acid-dependent cell wall loosening, which in turn permits cell growth [29, 30]. In our study, changes in the content of IAA in etiolated mesocotyls at different growth periods coincided with the mesocotyl growth curve (Fig. 2b) and varied in different zones of the mesocotyl (Fig. 2c). Mesocotyl segments grew quickly in the first 24 h in the presence of IAA and slowed down until its growth was ceased. This effect was probably due to a loss of turgor pressure caused by the dilution of vacuolar contents [31] or due to reaching the final sizes of mesocotyl cells [32]. We also found that the cellulose content of mesocotyl segments grown in vitro was decreased by exogenous IAA [33] or gibberellin [34]. Similarly, almost all protein spots in the 2-DE gels declined in abundance for mesocotyl segments grown in vitro (data not shown), i.e., lacking net protein synthesis, as previously reported [35]. This finding could be explained by the fact that in intact tissues, the balance between synthesis and hydrolysis is actively maintained, whereas in excised tissues, the synthesis is greatly slowed or inhibited, and hydrolytic events predominate.

We found that there was an obvious change in the protein patterns with the growth of the maize mesocotyl (Figs. 4, 5, 6 and 7); that is, the mesocotyl at different periods had a distinct set of DAPs. To obtain reliable proteomic data, the DAPs identified here by Mascot search have manually been checked by referring to its MW and p*I* in 2-D gels and by searching the nonredundant database of UniProtKB for each possible DAPs. Due to the inherent limitations of 2-DE, the DAPs identified here may represent a small number of proteins in the mesocotyl proteome. Using a more robust gel-free (e.g., iTRAQ-based) proteomic analysis will reveal comprehensive proteome changes during maize mesocotyl growth.

In 48-h-old mesocotyls, most DAPs (79%) were heat shock proteins, stress proteins and proteins related to protein biogenesis and degradation, and the other proteins (21%) were storage proteins, oxidation/reduction proteins and carbohydrate biogenesis-related proteins. These DAPs were mainly involved in cellular process, metabolic process and response to stimulus (Fig. 6, Additional file 5: Figure S2a) and required for protein and carbohydrate metabolism to prepare raw materials and energy at the initial growth of the maize mesocotyl. Particularly, the 60S acidic ribosomal protein P3 (spots 67, 89) of the structural constituent of ribosome existed in highest abundance during the initial period than in the later periods, and its decreasing abundance was consistent with the increased activity of ribonuclease during maize mesocotyl growth [35].

In 84-h-old mesocotyls, the proportions of carbohydrate biosynthesis-related proteins, oxidation/reduction enzymes and cytoskeleton proteins among the DAPs significantly increased (Fig. 6, Additional file 5: Figure S2a). Cell growth at the tip of the etiolated mesocotyl is dependent on vacuole enlargement and the massive flux of endoplasmic reticulum (ER) and Golgi vesicles. The expression of V-ATPase genes was found to be positively correlated with cellular growth [11]. In rye, the cessation of coleoptile growth was associated with the downregulation of the subunit E of the V-ATPase [29]. Water flow into the expanding vacuole is driven by ion accumulation, which in turn is energized by the vacuolar H^+^-ATPase (V-ATPase) [29]. We found here that V-ATPase (spots 5 and 23 corresponding to catalytic subunits A and B, respectively) was highly accumulated during the rapid growth period of the mesocotyl. The microtubule and actin cytoskeleton mediates cell growth, division and morphogenesis [36]. We found that actin-7, existing in four spots (2, 3, 39 and 40), increased in abundance during the rapid growth period of the mesocotyl. Tubulin was also observed to highly accumulate in the rice coleoptile under anoxic conditions [37]. Moreover, cytochrome c oxidase subunit 5b-2 (spot 10), which is involved in mitochondrial electron transport, had a higher abundance during this period. However, a previous study showed that red light inhibited mesocotyl growth in dark-grown maize seedlings, along with the decreased activity of cytochrome reductase but without a detectable change in the activity of cytochrome oxidase [9].

The cell wall is a dynamic structure that consists of an intertwined network of polysaccharides, lignin, and proteins [33, 38]. Cellulose is the most abundant component in the walls of growing cells. Cellulose consists of a collection of β-1,4-linked glucan chains that interact with each other via hydrogen bonds to form a crystalline microfibril [39]. We found here that the cellulose content reached a maximum during the rapid growth period of the mesocotyl, indicating quick wall synthesis, and then decreased thereafter. Cellulose biosynthesis involves a large multisubunit complex consisting of at least three different cellulose synthase enzymes [40]. The complex localized in the plasma membrane as a rosette structure that transfers Glc from cytosolic UDP-Glc to produce multiple extracellular glucan chains that eventually coalesce into a cellulose microfibril [33]. Some *cellulose synthase-like genes* (*ZmCSL*) are regulated by light, whose expression changes in parallel with the growth rate of the mesocotyl [41]. Consistent with the changes in cellulose content (Fig. 3), we found that UDP-glucose 6-dehydrogenase (spot 9) accumulated at higher abundance during the rapid growth period of the mesocotyl and then gradually decreased.

In 132-h-old mesocotyls, cell wall-related proteins remarkably increased in number (taking up 14% of type III DAPs) (Fig. 6, Additional file 5: Figure S2a). Mesocotyl growth is closely related to cell wall extensibility or cell wall “loosening”, driven by the pressure of the protoplasm [1]. Wall extensibility can be accomplished by one or more wall-localized enzymes that catalyze the formation or breakage of cell wall bonds, especially the hydrolysis of cell wall glucans, in particular xyloglucan [34, 42]. Accordingly, we found that xyloglucan endoglucosylase/hydrolases (spots 22, 28, 88) began to accumulate during the rapid growth period and significantly increased with mesocotyl growth.

Moreover, hydrogen peroxide plays a key role in the modulation of extension growth by decreasing wall extensibility through the peroxidase-mediated oxidative cross-linking of cell wall glycoproteins and polysaccharides [23, 43]. Red light suppressed the mesocotyl growth of maize, resulting in a 40% increase in the level of peroxidase in mesocotyl walls [44]. Here, we found that peroxidase, existing in multiple spots (1, 15, 34, 43, 47) during the slow growth period, was increased in both abundance and enzyme activity, especially at the maturation part of the mesocotyl (Fig. 6). This result was consistent with previous results showing a negative correlation between wall peroxidase activity and wall growth [45, 46].

Additionally, the mRNA levels of six DAPs were partly correlated with protein abundance, and their gene expression occurred earlier than their protein accumulation (Fig. 7). The transcript/protein discordance has been well documented in maize [47], possibly because the protein life-time is far shorter than mRNA life-time and/or these transcripts are not fully translated.

## Conclusions

Until now, there is still a lack of omics studies on the growth of maize mesocotyl. In this study, we explored maize mesocotyl growth in vivo and in vitro at physiological and proteomic levels. The growth of maize mesocotyl, accompanied by changes in IAA content and cell wall cellulose, contributed greatly to seed germination and early seedling establishment. The protein patterns on 2-D gels differed with mesocotyl growth, and a total of 88 DAPs were associated with etiolated mesocotyl growth. At different periods of mesocotyl growth, a specific set of DAPs were involved in various biological processes and underlie the cellular and physiological activities. These results provide new information for understanding the growth of maize mesocotyl and the cultivation of maize mesocotyl with deep-sowing tolerance.

## Methods

### Plant material and growth conditions

Maize hybrid Zhengdan 958, currently grown widely in China, was used in the present study. Mature maize seeds were purchased from the Henan Qiule Seed Industry Science and Technology Company, Ltd. (Zhengzhou, China). The seeds were surface-sterilized with 0.1% sodium hypochlorite and rinsed thoroughly. After imbibition for 12 h in water, the imbibed seeds were germinated in growing medium (Pindstrup Substrate, 0–6 mm, pH 6.0, Pindstrup Mosebrug A/S, Denmark) in plastic pots (25 × 19 × 30 cm) at sowing depths of 5 cm, 10 cm and 15 cm, respectively. The pots were well watered and placed in a greenhouse with a photoperiod of 16 h light (80–100 μE m^− 2^ s^− 1^, 28 °C) and 8 h dark (22 °C). After 14 days, the seedling emergence rate, mesocotyl and coleoptile length were measured. All of the above experiments were carried out with at least three independent biological replicates.

Additionally, the imbibed seeds were germinated on wet papers in darkness at 25 °C for a week. For mesocotyl growth in vitro*,* 10 mm segments were excised under dim green light from the apical region of the dark-grown 84 h-old mesocotyls downwards. Twenty segments were incubated for 24 h in the dark (25 °C) in MS medium (pH 6.0) plus 1% sucrose, with or without IAA (10 μM or 100 μM) [48]. The mesocotyls grown in vivo or in vitro were sampled at different times for various analyses.

### Physiological and biochemical analysis

For biomass and moisture content assays, the fresh and dry weights (after drying at 60 °C for 48 h) of mesocotyl samples were determined on a precision balance, and the RWC was calculated [49].

The cellulose content was determined using the anthrone-sulfuric acid method [50]. The mesocotyl sample (0.02 g dry weight) was hydrolyzed in 6 ml 60% H_2_SO_4_ for 30 min, and then 2 ml of the hydrolysate was mixed with 0.5 ml 2% anthrone reagent and 5 ml H_2_SO_4_ and incubated for 10 min. After centrifugation of the mixture, the absorbance at 620 nm was read, with cellulose as a standard.

The levels of IAA in maize mesocotyls were quantified by high-performance liquid chromatography-tandem mass spectrometry (HPLC-MS/MS) [51]. Mesocotyl samples (1.0 g fresh weight) were ground in a mortar with liquid N_2_ and then homogenized in 10 ml isopropanol/hydrochloric acid buffer. The extract was shaken at 4 °C for 30 min, and then 20 ml dichloromethane was added and shaken at 4 °C for 30 min. After centrifugation at 13,000 rpm for 5 min at 4 °C, the organic phase was extracted and dried under N_2_. The resulting dry pellets were resuspended in 200 μl methanol plus 0.1% methane acid and filtered with a 0.22-mm filter membrane. The filtrate was used for HPLC-MS/MS with a ZORBAXSB-C18 (Agilent Technologies) column (2.1 × 150; 3.5 um) at 30 °C. The sample injection volume was 2 μl. The mobile phase: A: B = (methanol/0.1% formic acid): (water/0.1% formic acid); elution gradient: 0–2 min, A = 20%; 2–14 min, An increased to 80%; 14–15 min, A = 80%; 15.1 min, A decreased to 20%; 15.1–20 min, A = 20%. The MS conditions were as follows: the pressures of the air curtain, nebulizer, and aux gas were 15, 65, and 70 psi, respectively; the spray voltage was 4500 V; and the atomizing temperature was 400 °C.

### Light microscopy

Mesocotyl samples were fixed in the FAA solution for 48 h, maintained in 50% ethanol for 24 h and then dehydrated in a series of alcohols and embedded in paraffin wax. Serial sections were longitudinally cut along the tangential direction with a sliding microtome at 10- to 15-μm thick, stained with safranin-fast green, and mounted in Canada balsam [52]. Ten to twelve slides were prepared from each sample. The specimens were observed using a Phoenix PH50 light microscope and recorded and digitized using ToupView × 86 software (ToupTek Photonics; China).

### Protein extraction and quantitation

Mesocotyl proteins were extracted as described previously [53]. The mesocotyls were pulverized in a mortar and pestle with liquid N_2_ and then homogenized with cold acetone plus 5 mM DTT. The acetone-washed dry tissue pellets were subjected to protein extraction in an SDS buffer containing 1% SDS, 0.1 M Tris-HCl (pH 6.8), 2 mM EDTA-Na_2_, 20 mM DTT and 2 mM PMSF. The resulting protein extract was precipitated with an equal volume of cold 20% TCA/acetone. The protein precipitates were washed twice with 80% acetone, air-dried and dissolved in Laemmli SDS buffer for SDS-PAGE or in 2-DE rehydration solution containing 7 M urea, 2 M thiourea, 2% CHAPS, 20 mM DTT, 0.5% IPG buffer (pH 4–7, GE Healthcare) for isoelectric focusing (IEF) [54]. Alternatively, the protein precipitates were dissolved in the 2-DE rehydration solution without the IPG buffer to avoid interference with the assay [55]. After protein quantitation, IPG buffer was supplemented into protein samples to a final concentration of 0.5% for IEF.

### SDS-PAGE, immunoblotting and peroxidase (POD) activity staining

SDS-PAGE was performed on a Laemmli gel system (5% stacking gel and 12.5% resolving gel). After electrophoresis, the gels of mesocotyl proteins were stained with 0.1% Coomassie brilliant blue (CBB) R-350 overnight and destained in 7% acetic acid until a clear background was obtained. The gels were photographed using a DSLR camera (Nikon D7000) in automatic mode.

For immunoblotting, the protein gels were electrophoretically transferred onto a polyvinylidene difluoride membrane (Hybond-P, GE healthcare) in a transfer buffer (20% v/v methanol, 48 mM Tris, 39 mM glycine) for 20 min at 15 V on a semi-dry electrophoretic transfer cell system (Trans-Blot, Bio-Rad, USA). After incubation in 5% skimmed milk in TBST buffer (50 mM Tris-HCl, pH 7.5, 0.15 M NaCl, 0.1% Tween-20) for 1 h, the membrane was incubated with anti-actin and anti-tubulin polyclonal antibodies (A01050, A01030, Abbkine; USA) in TBST buffer (1:5000 dilution) for 1 h, respectively, and subsequently incubated with POD-conjugated goat anti-mouse IgG (CW0102S, CWBIO, China) (1:5000 dilution) for 1 h. After extensive washing, the blots were detected with chemiluminescent HRP substrate for 1–5 min (Immobilon™ Western, Millipore Corporation, Billerica, USA), and the images were captured with a chemiluminescence/fluorescence image analysis system (Tanon 5200, Shanghai, China).

For POD isozyme activity staining, the protein gels were stained with 3,3′-diaminobenzidine at room temperature for 10 min [56]. The stained gels were photographed, and the digital images were processed and analyzed using PDQuest8.0 software (Bio-Rad, USA).

### 2-DE and mass spectrometry

IEF was performed with a PROTEAN IEF CELL system (Bio-Rad, USA) using 11 cm linear pH 4–7 IPG strips (Bio-Rad, USA). The proteins (600 μg in 220 μl) were passively loaded onto the strips, and IEF and subsequent SDS-PAGE were performed as previously described [57]. After electrophoresis, the protein gels were stained using CBB R350 and photographed using a DSLR camera (Nikon D7000) in automatic mode.

The gel images were analyzed using PDQuest 8.0 software (Bio-Rad, USA) to compare statistically significant differences in protein accumulation in different samples. The spots were automatically detected and edited manually to improve accuracy. Matching was automatically obtained and manually checked. The relative volume parameter (%Vol) was used to evaluate protein level differences between gels. The %Vol value of spots in fluoxetine-treated samples was normalized to the corresponding value in the control sample, and the obtained data were analyzed for significant differences using a one-way analysis of variance model based on three biological replications. Protein abundance was determined by taking the average normalized standardized spot peak area of all spots in 2-D gels. The spots with at least two-fold changes with statistically significant (t-test with *p* < 0.05) and reproducible changes in abundance among the three growth periods of mesocotyls were selected for protein identification. A MALDI-TOF/TOF mass spectrometer (ABI 5800, Applied Biosystems, CA, USA) was used in reflection mode to analyze the peptides from the tryptic digest as described previously [57]. The spectra were acquired in the positive ion mode and automatically submitted to Mascot 2.2 (http://www.matrixscience.com) for identification against the NCBInr database (February 17, 2017; species, *Zea mays*; 279,566 sequences). The search parameters were as follows: type of search: MALDI-TOF ion search; enzyme: trypsin; fixed modifications: carbamidomethyl (C); variable modifications: acetyl (protein N-term), deamidated (NQ), dioxidation (W), oxidation (M); mass values: monoisotopic; protein mass: unrestricted; peptide mass tolerance: ±100 ppm; fragment mass tolerance: ±0.3 Da; max missed cleavages: 1.

To avoid random matches, only ions with individual scores above the value indicated by MASCOT to identify or determine extensive homology (p < 0.05) were considered for protein identification. Proteins with significant MASCOT scores (> 38) and at least three peptide sequences confirmed by MS/MS were considered positively identified (p < 0.05). Unambiguous identification was judged by the number of MASCOT scores, matched peptide sequences, sequence coverage, MW and *p*I. Moreover, a standard protein BLAST was run against the UniProtKB (http://www.UniProt.org/blast/) to search for homologous ‘uncharacterized’ or ‘hypothetical’ proteins.

### Bioinformatics analysis

Database searches using individual tryptic fragments were performed using BLAST searches at NCBI (http://www.ncbi.nlm.nih.gov/blast). The identified proteins were functionally classified according to the annotations in the UniProtKB database (https://www.UniProt.org/). Subcellular locations of the identified proteins were determined according to the annotation in UniProtKB or predicted at Plant-mPLoc server (http://www.csbio.sjtu.edu.cn/bioinf/plant-multi/). A Venn diagram was constructed using online server (http://bioinformatics.psb.ugent.be/webtools/Venn/). The heatmap was constructed using Microsoft Excel. The DAPs were annotated using Blast2GO software 5.0 (https://www.blast2go.com/). Protein sequences were compared against the SwissProt database using a public NCBI Blast service (QBlast), and all programs were executed using default configurations. The meaningful matches from the Blast2GO analysis were annotated to GO categories (cellular component, molecular function, and biological process).

### RT-qPCR

The total RNA of maize mesocotyls was extracted using RNA-*Solv*® reagent (Omega Bio-Tek, USA) according to the manufacturer’s instructions. The purity and concentration of total RNA were determined by a NanoDrop One spectrophotometer (Thermo Scientific, USA). Total RNA (10 μg) was converted to cDNA using the FastKing RT Kit with gDNase (TIANGEN, China). The gene-specific primers (Additional file 3: Table S3) were designed using Primer Premier 5.0 and synthesized at the Biomed Cooperation (Beijing, China), with the ubiquitin gene as a loading control. RT-qPCR was performed using the StepOnePlus™ Real-Time PCR Instrument Thermal Cycling Block (Applied Biosystems). The PCR cycle was run as follows: 95 °C for 3 min, followed by 35 cycles at 95 °C for 3 s and 58 °C for 30 s. The relative expression level of each gene was calculated from the cycle threshold (Ct) value as 2^−ΔCt^ (ΔCt  =  each corresponding Ct value−the minimum Ct value), with the maximum expression level (set to 1.0) of each gene as a control.

## Supplementary information

**Additional file 1:** **Table S1.** Consensus analysis and relative abundance changes in mesocotyl proteins resolved in 2-DE. The relative abundances (volumes) of the spots were determined using PDQuest8.0 software.

**Additional file 2:** **Table S2.** Summary of the DAPs identified in maize mesocotyl.

**Additional file 3:** **Table S3.** Primers used in RT-qPCR.

**Additional file 4:** **Figure S1.** The 2-DE profiles of maize mesocotyl at 48 h, 84 h and 132 h. Maize mesocotyl proteins (600 μg) were resolved by IEF using 11 cm pH 4–7 IPG dry strips. Secondary SDS-PAGE was carried out on a 12.5% resolving gel. The proteins were visualized using CBB R350 staining. Spots of relatively abundant proteins in the maize mesocotyl at 48 h, 84 h and 132 h with at least a two-fold change in abundance are indicated with a red line.

**Additional file 5:** **Figure S2.** Pie charts of the DAPs identified from the maize mesocotyl. A, Classification of the DAPs based on molecular function. B, Classification of the DAPs based on subcellular localization.

## Data Availability

All data have been presented in the main paper or additional supporting files.

## Abbreviations

2-DE: Two-dimensional electrophoresis
CBB: Coomassie brilliant blue
Ct: Cycle threshold
DAPS: Differential abundance proteins
ER: Endoplasmic reticulum
GO: Gene ontolgy
HSP: Heat shock protein
IAA: Indole-3-acetic acid
IEF: Isoelectric focusing
MALDI-TOF/TOF: Matrix-assisted laser desorption/ionization time-of flight/time-of-flight
MS: Mass spectrometry
POD: Peroxidase
