# Supplementary material for: 2-DE-based proteomic analysis of protein changes associated with etiolated mesocotyl growth in Zea mays
Source: BMC Genomics. 2019 Oct 22;20:758. doi: 10.1186/s12864-019-6109-z (PMC6805590; doi:10.1186/s12864-019-6109-z)
Supplement: Supplementary file 2 — Additional file 2: Table S2. Summary of the DAPs identified in maize mesocotyl. [file 12864_2019_6109_MOESM2_ESM.docx]

**Table S2** Summary of the DAPs identified in maize mesocotyl

| **Spot** | **NCBI** | | **UniProtKB** | | **Mass (kDa)** | ***p*I** | **Coverage (%)** | | **Score** | **Matched sequences** | |
| --- | --- | --- | --- | --- | --- | --- | --- | --- | --- | --- | --- |
|  | **Protein** | **Accession** | **Protein** | **Accession** |  |  |  |  |  |  |  |
| 1 | Peroxidase 12 | ONM15606.1 | Peroxidase | A0A1D6E530 | 35.97 | 5.35 | 33 | 233 | | | GPQGNVGEIR; SSVDAALQQNVR; DAVSLSGGPSFTVPLGR; CSSFGDVAGPATDDVTR; GLPPNVGLQQEAVQLVEDI; LLFHDCFPQGCDASILLDNGER |
| 2 | Tubulin alpha-4 chain | AQK63225.1 | Tubulin alpha chain | A0A1D6GIG4 | 50.07 | 4.89 | 27 | 164 | | | FDLMYAK; EDAANNFAR; EIVDLCLDR; QLFHPEQLISGK; SLDIERPTYTNLNR; AVFVDLEPTVIDEVR; TIGGGDDAFNTFFSETGAGK; FDGALNVDVNEFQTNLVPYPR |
| 3 | tubulin alpha-6 chain | NP_001105588.1 | Tubulin alpha chain | C0PKT5 | 50.20 | 4.92 | 19 | 167 | | | EIVDLCLDR; QLFHPEQLISGK; SLDIERPTYTNLNR; AIFVDLEPTVIDEVR; IHFMLSSYAPVISAEK; FDGAINVDVTEFQTNLVPYPR |
| 4 | Phosphoglycerate mutase-like protein | ONM19131.1 | Phosphoglycerate mutase-like protein | A0A1D6EG37 | 31.34 | 5.95 | 28 | 90 | | | HFANCELR; FMDWLWTR; IELVITSPLLR; TMQTAVGVFGGEK; NPTGLDVPSDIADKK; MELGATTALYPLHR |
| 5 | vacuolar proton pump3 | AQK60306.1 | Putative ATPase, V1 complex, subunit A protein | A0A1D6QS42 | 65.58 | 5.43 | 23 | 291 | | | FDPDFIDIR; LYDDLTTGFR; LAADTPLLTGQR; FTMLQTWPVR; VGHDNLIGEIIR; YSNSEAVVYVGCGER; NIIHFNTLANQAVER; NIIHFNTLANQAVER; VTTFEDSEKESEYGYVR; LEGDSATIQVYEETAGLMVNDPVLR |
| 6 | Ran-binding protein 1 homolog | ONL96963.1 | Ran-binding protein 1 homolog a | A0A1D6JZE5 | 25.06 | 4.74 | 44 | 209 | | | EEMFAIR; FGSVENCK; FKDTVEEIAEQQGK; SEEKENDEVSTAASDLAQK; LEEVAVTTGEEDEDALLDMK; AEPAEHRPAEEEEAAAAGEDEDTGAQVAPIVK |
| 7 | Calreticulin-2 | ONM21222.1 | Calreticulin-2 | A0A1D6EN35 | 45.50 | 4.49 | 22 | 124 | | | FEDGWESR; FEDGWESR; GIQTSEDYR; TFAEETWGK; YIGIELWQVK; FYAISAEYPEFSNK; SGTLFDNIIITDDPALAK; APMIDNPDFKDDPYIYAFDSLK |
| 8 | DUF538 family protein | AQK82674.1 | DUF538 family protein | B4FMB1 | 17.50 | 5.35 | 63 | 347 | | | STGFVWLR; EVTAFVEPGR; QAAGLTHTFGSIGK; SGAEVHVGHELCER; SGAEVHVGHELCER; AFPVTAFQLEPAAAEGDHKEEAEDAAAAAAN |
| 9 | UDP-glucose 6-dehydrogenase | AQK62747.1 | UDP-glucose 6-dehydrogenase | B7ZYX8 | 53.53 | 5.71 | 27 | 545 | | | LAANAFLAQR; NLFFSTDVEK; AADLTYWESAAR; AADLTYWESAAR; DVYAHWVPEDR; ILTTNLWSAELSK; CPDIEVVVVDISKPR; IFDNMQKPAFVFDGR; AQISIYDPQVTEDQIQR; GINYQILSNPEFLAEGTAIEDLFKPDR |
| 10 | Cytochrome c oxidase subunit 5b-2 mitochondrial | AQK96554.1 | Cytochrome c oxidase subunit 5b-2 mitochondrial | K7V763 | 18.05 | 4.94 | 19 | 37 | | | FDMDSPVGPFGTK; VIGDGGDPDGHDDEDDGHH |
| 11 | Ankyrin repeat domain-containing protein 2A | AQK57769.1 | Ankyrin repeat domain-containing protein 2A | B4G137 | 36.40 | 4.50 | 11 | 92 | | | NTPLHYAAGYGR; ALHFACGYGELK; ALDGGADKDEEDSEGR |
| 12 | Putative USP family protein | AQK95807.1 | Putative USP family protein | K7V2N6 | 27.34 | 5.24 | 25 | 116 | | | AGDHLLLLHVIK; EALQWAATNLLR; LCQVIHDTPLSCLVIGSR; YGAKPDMETLDLLNTTATQK |
| 13 | Golgi associated protein-like protein | ONM06916.1 | Alpha-1,4-glucan-protein synthase | B4FQX1 | 41.72 | 5.75 | 22 | 268 | | | ASNPFVNLK; LGTIDPYFVK; DINALEQHIK; VPEGFDYELYNR; YIYTIDDDCFVAK; AGTVTVPGSSTPSTPLLKDELDIVIPTIR |
| 14 | PLAT domain-containing protein 3 | ONM16985.1 | PLAT domain-containing protein 3 | C4J9R0 | 22.00 | 6.41 | 13 | 67 | | | GNLDIFSGR; DLAGWGGLMGAGHDYYER |
| 15 | peroxidase 39 precursor | NP_001149755.1 | Peroxidase | B6THU9 | 35.81 | 7.59 | 27 | 364 | | | DAAPNLTLR; TFDLSYYR; THFHDCFVR; AHIPHAPDVASTLLR; GLFQSDAALITDAASK; DSVGVIGGPFWSVPTGR; ECPGVVSCADIVALAAR |
| 16 | Ribulose-phosphate 3-epimerase | ONM56214.1 | Ribulose-phosphate 3-epimerase | A0A1D6I841 | 26.73 | 5.54 | 21 | 84 | | | AGASGFTFHIEVAR; IAPSMLSSDFANLASEAER; AYLDCHLMVTKPSDYVEPFGK |
| 17 | germin-like protein 2 precursor | NP_001140856.1 | Germin-like protein subfamily T member 1 | B4FRS8 | 23.89 | 5.89 | 18 | 228 | | | AGETFVIPR; GELLVGIVGSLDSGNR; VDFAPGGTNPPHVHPR |
| 18 | NADPH:quinone oxidoreductase | ONM34402.1 | NADPH:quinone oxidoreductase | A0A1D6N0U0 | 22.21 | 6.15 | 23 | 140 | | | VLLSLQAFTLR; FDSDGNLVDAEIR; AAAEVCEDSIPGLR |
| 19 | NAD(P)H dehydrogenase (quinone) FQR1 | AQK99941.1 | Minor allergen Alt a 7 | B4FWD0 | 21.60 | 5.95 | 46 | 315 | | | AFFDATGGLWR; VYVVYYSMYGHVGK; WPSEVELEHAFHQGK; WPSEVELEHAFHQGK; LFGMDQVQGGSPYGAGTFAADGSR; MGAPPKPDVPVITPQELAEADGILFGFPTR |
| 20 | Profilin-1 | AQL00684.1 | Profilin | A0A1D6GCC8 | 9.69 | 5.14 | 36 | 54 | | | DFDEPGFLAPTGLFLGPTK |
| 21 | 12-dihydroxy-3-keto-5-methylthiopentene dioxygenase 3 | ONL93870.1 | 1,2-dihydroxy-3-keto-5-methylthiopentene dioxygenase | A0A1D6JPD5 | 26.39 | 5.08 | 28 | 144 | | | DQDDQWIR; LSELGILSWR; FTLDSDNYIK; YCLEGSGYFDVR; NFFEEHLHTDEEIR |
| 22 | xyloglucan endotransglycosylase/hydrolase protein 8 precursor | ACG43095.1 | Xyloglucan endotransglucosylase/hydrolase | B4FTH5 | 32.24 | 5.53 | 24 | 172 | | | FTTEGNVR; FPQGFPAECSR; VDWSAAPFAVSYR; ADYNAQGQQVTSLILTQQSGGAFSSR |
| 23 | vacuolar proton pump2 | AQL04083.1 | Vacuolar proton pump2 | A0A1D6P248 | 55.45 | 5.12 | 22 | 165 | | | YQEIVNIR; FVTQGAYDTR; AVVQVFEGTSGIDNK; QIYPPINVLPSLSR; VTLFLNLANDPTIER; GYPGYMYTDLATIYER; AVVGEEALSSEDLLYLEFLDK; AVVGEEALSSEDLLYLEFLDKFER |
| 24 | pathogenesis-related protein 10 | AAY29574.1 | Pathogenesis-related protein 10 | Q29SB6 | 17.05 | 5.39 | 40 | 191 | | | AAVMDWHTLAPK; LLPGVEVKDEIAK; ASANSWTLEIASPVAPQR; VASHVVASAQPVEGDGGVGSVR |
| 25 | proteasome subunit alpha type 1 | NP_001149085.1 | Proteasome subunit alpha type | B6TAJ3 | 30.28 | 5.19 | 41 | 440 | | | LSSSNCTVAIVGR; VADHAGVALAGLTADGR; EDGTIEPFEMIGAAR; FQGYNDYTPEQLIK; NQYDTDVTTWSPQGR; KEDGTIEPFEMIGAAR; NQYDTDVTTWSPQGR; NECINHSFVYEAPLPVSR; SSTHAVLAAVNKPASELSSYQR |
| 26 | fructokinase-2 | NP_001105211.1 | Fructokinase-2 | A0A1D6LDR6 | 28.28 | 5.98 | 28 | 268 | | | EFMFYR; EGILSIWK; DDSIFHNEEK; VSDDEVAFLTR; NVLSLWFDGLK; AAGVLCSYDPNVR; VFHYGSISLISEPCR |
| 27 | Metacaspase-6 | ONM37196.1 | Metacaspase-6 | A0A1D6N9H5 | 46.42 | 5.75 | 6 | 111 | | | GCYNDVDR; FGFDEADIR; SAPQPTGANIR |
| 28 | putative xyloglucan endotransglucosylase/hydrolase protein 32 | ONL92742.1 | Xyloglucan endotransglucosylase/hydrolase | B4FHS5 | 33.85 | 6.64 | 23 | 184 | | | NGYFGVSIK; YAAASAPFFPDR; VQPGYTAGVNTAFYLSNNELYPGR; HDEIDMELLGTVPGEPYTLQTNVYVR |
| 29 | profilin-5 | NP_001105622.1 | Profilin-5 | Q9FR39 | 14.22 | 4.59 | 32 | 203 | | | LGDYLIEQGF; YMVIQGEPGVVIR; YMVIQGEPGVVIR |
| 30 | profilin-5 | NP_001105622.1 | Profilin-5 | Q9FR39 | 14.22 | 4.59 | 32 | 216 | | | LGDYLIEQGF; YMVIQGEPGVVIR; YMVIQGEPGVVIR |
| 31 | wound/stress protein precursor | NP_001149031.2 | Wound/stress protein | K7U5W7 | 19.94 | 5.26 | 4 | 73 | | | SNLDIFSGR |
| 32 | anther-specific proline-rich protein APG precursor | NP_001149080.1 | Anther-specific proline-rich protein APG | B4F9E3 | 40.22 | 5.31 | 30 | 267 | | | TLYSYGAR; DETGQQLGQR; ANYPPYGIDFAAGPTGR; APGSHGLTVTNQGCCGVGR; STDGTTCVPQINGAIDIFNR; NEYLFWDAFHPTEAANILVGR |
| 33 | Peroxidase 12 | ONM15606.1 | Peroxidase | A0A1D6E530 | 35.97 | 5.35 | 39 | 669 | | | GPQGNVGEI; SSVDAALQQNVR; GVMLPSDQGLVSDPR; DAVSLSGGPSFTVPLGR; CSSFGDVAGPATDDVTR; VHAACGPTVSCADITVLATR; GLPPNVGLQQEAVQLVEDIR; LLFHDCFPQGCDASILLDNGER |
| 34 | Survival protein SurE-like phosphatase/nucleotidase | AQK97772.1 | Survival protein SurE-like phosphatase/nucleotidase | A0A1D6G329 | 44.85 | 5.08 | 10 | 252 | | | YEGLDEDIDLR; ETIAASSVDFTGAK; RYEGLDEDIDLR; AFETSGTPVDCVSLALSGR |
| 35 | ripening-related protein 3 | XP_008644746.1 | Ripening-related protein 3 | A0A1D6GNR3 | 21.41 | 6.50 | 34 | 288 | | | FYDDSKK; DHNFEPPCR; VVALSTGWYNGGSR; VVALSTGWYNGGSR; YTTYGCSPPVTGSTR; AVLTLNSFAEGGDGGGAAACTGK |
| 36 | pathogenesis-related protein 10 | AAY29574.1 | Pathogenesis-related protein 10 | Q29SB6 | 17.05 | 5.39 | 40 | 291 | | | AAVMDWHTLAPK; LLPGVEVKDEIAK; ASANSWTLEIASPVAPQR; VASHVVASAQPVEGDGGVGSVR |
| 37 | putative 2-oxoglutarate-dependent dioxygenase AOP1 | ONM16724.1 | Putative 2-oxoglutarate-dependent dioxygenase AOP1 | B4FN73 | 32.71 | 5.32 | 23 | 245 | | | AEIPVIDLR; LAGSSPDESAR; ALFDLPDDAK; ALFDLPDDAKR; YKPFNYDDYR; VSAPEALVDAGHPR; VTGHGAPAGLLADMK |
| 38 | Putative translation elongation factor family protein | AQL00681.1 | Putative translation elongation factor family protein | A0A1D6GCC4 | 92.36 | 5.83 | 13 | 250 | | | GGGQVIPTAR; GVQYLNEIK; EGALAEENMR; DSVVAGFQWASK; EQMTPLSEFEDKL; AFLPVIESFGFSSQLR; GHVFEEMQRPGTPLYNIK; DLQEDFMGGAEIIVSPPVVSFR |
| 39 | Actin-7 | AQK95630.1 | Actin-7 | A0A1D6FW13 | 50.54 | 5.94 | 25 | 510 | | | AGFAGDDAPR; GYSFTTSAER; EEYDESGPAIVHR; IWHHTFYNELR; SYELPDGQVITIGAER; LAYIALDYEQELETAK; VAPEEHPVLLTEAPLNPK; DLYGNIVLSGGSTMFPGIADR |
| 40 | Actin-7 | ONM19688.1 | Actin-7 | A0A1D6EHT1 | 66.14 | 5.47 | 14 | 371 | | | AGFAGDDAPR; GYSFTTSAER; AEYDESGPGIVHR; SYELPDGQVITIGAER; VAPEEHPVLLTEAPLNPK; DLYGNIVLSGGTTMFPGIADR |
| 41 | UMP-CMP kinase family protein | AQK56201.1 | UMP-CMP kinase | A0A1D6QDC6 | 25.93 | 5.64 | 37 | 369 | | | FLIDGFPR; SENDKFLIDGFPR; IDAAKPIPEVFEDVK; KIDAAKPIPEVFEDVK; TFVDSTLPVIEHYNSK; GTVVDAAPAVVAEVTENMLGGK; ISPAFVLFFDCSEEDMEKR |
| 42 | APx1 - Cytosolic ascorbate peroxidase | NP_001150192.1 | APx1-cytosolic ascorbate peroxidase | B6TM55 | 27.46 | 5.55 | 43 | 471 | | | AFFDDYK; SCAPLMLR; EDKPQPPPEGR; LAWHSAGTFDVSSR; ALLSDPVFRPLVEK; NYPTVSAEYSEAVEK; QMGLSDQDIVALSGGHTLGR; SGFEGAWTTNPLVFDNSYFK |
| 43 | Phosphoenolpyruvate carboxylase family protein | ONM18098.1 | Phosphoenolpyruvate carboxylase family protein | A0A1D6ECS1 | 13.85 | 5.01 | 29 | 230 | | | KESGSDIVIVAR; QAISHDEALWR; QAISHDEALWR; TPILSPAELEEIGFR |
| 44 | cysteine synthase | NP_001105469.2 | Cysteine synthase | B8A377 | 34.23 | 5.67 | 44 | 621 | | | YLSSVLFQSIK; LFVVVFPSFGER; LTLTMPASMSMER; EGLLVGISSGAAAAAAVR; AFGAELVLTDPLLGMK; IDGLVSGIGTGGTITGTGR; DVTELIGNTPLVYLNK; TPNSYILQQFENPANPK; LYGVEPVESAVLNGGKPGPHK |
| 45 | Dihydropyrimidine dehydrogenase (NADP(+)), chloroplastic | PWZ24913.1 | Dihydropyrimidine dehydrogenase (NADP(+)) chloroplastic | B4FZU9 | 46.04 | 6.07 | 29 | 258 | | | ATVPVWAK; AAWHELIER; AFDEGWGAVIAK; EHNFSSIEEFR; ILIGSIMEEYNK; MTPNITDITQPAR; GASLPYFTTHTDLVHR; TLRPEPCVEGYSTPGGYSAR |
| 46 | 4-hydroxy-7-methoxy-3-oxo-3,4-dihydro-2H-1,4-benzoxazin-2-yl glucoside beta-D-glucosidase 1, chloroplastic | P49235.1 | 4-hydroxy-7-methoxy-3-oxo-3,4-dihydro-2H-1,4-benzoxazin-2-yl glucoside beta-D-glucosidase 1, chloroplastic | P49235 | 64.54 | 6.23 | 17 | 239 | | | FSISWPR; YGIVYVDR; IGLAFDVMGR; SIVEDYTYFAK; ETPLPMEAALNDYKR; LAGSYNMLGLNYYTSR; SWDINLGWFLEPVVR; VGSQNGVQMLSPSEIPQR |
| 47 | Golgi associated protein-like protein | ONM06916.1 | α-1,4-glucan-protein synthase | B4FQX1 | 41.72 | 5.75 | 32 | 444 | | | LGTIDPYFVK; VPEGFDYELYNR; YIYTIDDDCFVAK; GTLFPMCGMNLAFDR; DLIGPAMYFGLMGDGQPIGR; NLLSPSTPFFFNTLYDPYR; AGTVTVPGSSTPSTPLLKDELDIVIPTIR |
| 48 | Legumin-like protein | NP_001105062.1 | Legumin-like protein | Q84TL7 | 38.05 | 5.79 | 30 | 559 | | | VQVVGPDGVR; ASSSPLPAPSPR; VEGGFLFIVPR; RLDSEIFFAPPSN; AISAEVLQASFNTTPEMEK; AWDLPEADAAALVSSQPASGIVR; AWDLPEADAAALVSSQPASGIVR; DREGVALNCLEAPLDVDIPGGGR |
| 49 | Malate dehydrogenase, cytoplasmic | NP_001105603.1 | Malate dehydrogenase | A0A1R3MB28 | 35.91 | 5.77 | 31 | 374 | | | TSTGEKPVR; MELVDAAFPLLK; IVQGLPIDEFSR; VLVVANPANTNALILK; FSSALSAASSACDHIR; VLVTGAAGQIGYALVPMIAR; ELVSDDEWLNGEFITTVQQR; ELVSDDEWLNGEFITTVQQR |
| 50 | UDP-arabinopyranose mutase 3 | AQK64294.1 | UDP-arabinopyranose mutase 3 | B4G039 | 41.74 | 6.11 | 29 | 335 | | | VPEGFDYELYNR; YIYTIDDDCFVAK; GTLFPMCGMNLAFDR; DLIGPAMYFGLMGDGQPIGR; NLLSPSTPFFFNTLYDPYR; NLLSPSTPFFFNTLYDPYR |
| 51 | Putative glutathione peroxidase | NP_001141210.1 | Glutathione peroxidase | Q6JAH6 | 18.62 | 6.59 | 34 | 287 | | | FLVDKEGR; AEYPIFDK; FKAEYPIFDK; YAPTTSPLSIEK; AASSTATSVHDFIVK; AEYPIFDKVDVNGSNAAPIYK |
| 52 | Cysteine proteinase 2 | ONM55302.1 | Cysteine proteinase 2 | A0A1D6I5B2 | 33.80 | 8.57 | 18 | 194 | | | FKNENVGVK; DWREDGIVSPVK; NSWGADWGDEGYFK; NSWGADWGDEGYFK; NSWGADWGDEGYFK; YNGGLDTEESYPYQGVNGICK |
| 53 | Glutathione S-transferase 3 | NP_001105111.2 | Glutathione S-transferase 3 | B4FK84 | 23.88 | 5.96 | 36 | 342 | | | VLDVYEAHLAR; LYGMPLSPNVVR; YASEGTDLLPATASAAK; GLDFEIVPVDLTTGAHK; YLAGDEFTLADANHASYLLYLSK |
| 54 | Isoflavone reductase homolog IRL | NP_001105699.2 | Isoflavone reductase-like1 | B4FD74 | 32.91 | 5.52 | 33 | 398 | | | LGHPTSALVR; EYVPEEAVLK; TFQDAGVTLLK; ILVVGGTGYLGR; YTTVDEYLNR; FFPSEFGLDVDR; GADVVISVLGSMQIADQSR; AVFVEEGDIATYTVLAADDPR |
| 55 | Putative translation elongation factor family protein | AQL00681.1 | Putative translation elongation factor family protein | A0A1D6GCC4 | 92.36 | 5.83 | 7 | 110 | | | GGGQVIPTAR; AFLPVIESFGFSSQLR; SQILSQEFGWDKDLAK |
| 56 | Caffeoyl-CoA O-methyltransferase 1 | NP_001152511.1 | Caffeoyl-CoA O-methyltransferase 1 | B6UF45 | 27.50 | 4.85 | 6 | 91 | | | VIYVAWR; NVESKILR; MVQPDLGR; DLLTQTARAAASSPR; QFTVTAAGGGATQTERVAAEMVR; GSSRHASGCGEQQGTGSNPMNSIDR; ANLQQEVARLLSESQNLK (N-terminal); YADNQPVVPWGPRFSR; CSSCMPSITDDFNEGVVDQQKLILDR |
| 57 | Enolase 1 | AQL02254.1 | Enolase 1 | A0A1D6NVW3 | 47.31 | 5.13 | 33 | 549 | | | FRAPVEPY; AVTITWVK (N-terminal); AGWGVMASHR; IEEELGDAAVYAGAK; VNQIGSVTESIEAVR; VQIVGDDLLVTNPTR; VVIGMDVAASEFFGEK; GAVPSGASTGIYEALELR; GNPTVEVDVGLSDGSYAR; YGQDATNVGDEGGFAPNIQENK |
| 58 | Thioredoxin family Trp26 | ONM41389.1 | Thioredoxin family Trp26 | B4FPP1 | 22.78 | 4.95 | 41 | 174 | | | SVFKPWEQR; VVALNESVPGSVK; SISVVGGADGTSPSR; DVVATIVYEVMPNPSDHK; FQGVANLTLHFSDNFGGDTTK |
| 59 | Thiamine thiazole synthase 2, chloroplastic precursor | NP_001105697.2 | Thiamine thiazole synthase, chloroplastic | B4G015 | 37.46 | 5.59 | 34 | 273 | | | HAALFTSTVMSR; LFNAVAVEDLIVR; MGPTFGAMMISGQK; VVVSSCGHDGPFGATGVK; ALGRPNAVDGTIPEVSPALR; EVVPGMIVTGMEVAEIDGAPR; RPAHLFLDELGVGYDEAEDYVVVK |
| 60 | Pathogenesis-related protein 2 | ABF81693.1 | Major latex protein 22 | Q19VG6 | 17.10 | 4.70 | 51 | 215 | | | VELVVEVK; IFPEQYK; ANDQVPDPDVIK; YTEAVPMLTFAK; VVSYSVVDGELADFYK; AEGEGGAVVSWAMEFDK; AEGEGGAVVSWAMEFDK |
| 61 | Protein disulfide isomerase 2 precursor | NP_001105755.1 | Protein disulfide-isomerase | A0A1D6F5C2 | 54.49 | 4.95 | 23 | 423 | | | IYIVGIFK; FIDASSTPR; GDASVEGPLIR; YEIQGFPTLK; VVVADNIHDVVFK; LLKPFDDLVVDSK; EFSGTEFTNFMELAEK; VVTFDNNPDNHPYLMK; FLIGDIEASQGAFQYFGLK |
| 62 | ABA-inducible gene protein | CAA31077.1 | Glycine-rich RNA-binding, abscisic acid-inducible protein | P10979 | 15.49 | 5.55 | 52 | 368 | | | AAADVEYR; DGGYGGGGGYGGR; EGGGGGYGGGGGYGGR; EGGGGGYGGGGGGWRD; REGGGGGYGGGGGYGGR; GFGFVTFSSENSMLDAIENMNGK; GFGFVTFSSENSMLDAIENMNGKELDGR |
| 63 | S-adenosylmethionine synthase 1 | ONM31848.1 | S-adenosylmethionine synthase | B4FIE9 | 43.20 | 5.59 | 20 | 315 | | | TAAYGHFGR; TIFHLNPSGR; FVIGGPHGDAGLTGR; EIGFTSDDVGLDADR; DDADFTWEVVKPLK; TQVTVEYVNEGGAMVPVR |
| 65 | 3-Oxoacyl-[acyl-carrier-protein] synthase I chloroplastic | AQL01354.1 | 3-oxoacyl-[acyl-carrier-protein] synthase I chloroplastic | K7V9P7 | 51.72 | 6.28 | 24 | 364 | | | RLDDCLR; GFSSEGYIDGK; LLAGESGIGPIDR; ALEAAGIAHGSKPMEK; DGFVMGEGAGVLVMESLEHAIK; VVITGMGLVSVFGNDVDAYYDR; AGVLVGTGMGGLTVFSDGVQNLIEK |
| 66 | NAD-dependent epimerase/dehydratase | ONM10954.1 | NAD-dependent epimerase/dehydratase | A0A1D6L9Y9 | 27.80 | 6.77 | 45 | 351 | | | TGQIVYNK; ALFSQITAR; IGAAGDVYVADIR; AFDLASKPEGVGTPTK; ADSATSRPTVLVTGAGGR; ELLVGKDDELLQTDTK; ADVAEVCVQALQYEEAK; LAPAVQGVDALIILTSAAPK |
| 67 | 60S acidic ribosomal protein P3-like | NP_001316611.1 | 60S acidic ribosomal protein P3 | A0A1D6LNJ7 | 12.04 | 4.45 | 21 | 188 | | | GVYTFVCR; QHSGEIEASAATPYELQR |
| 68 | Bowman-Birk type trypsin inhibitor | ONM30326.1 | Bowman-Birk type trypsin inhibitor | A0A1D6MM79 | 26.89 | 5.45 | 18 | 186 | | | YRGDPGPR; ECVETEDSR; TYPLTCSCFDR; DEERPWGECCDLAVCVK |
| 69 | Glycine-rich protein2 | AQK39923.1 | Glycine-rich protein2 | A0A1D6IV87 | 28.12 | 4.64 | 25 | 174 | | | YGQVIEAK; LFVGGLSYGTDDHSLR; LFVGGLSYGTDDHSLRDEFAK; GFGFITYTSSEEASAAITAMDGK; NDEIMDDLFKDDEPDSYANK |
| 70 | Glycine-rich RNA-binding protein 3 mitochondrial | ONM33600.1 | Glycine-rich RNA-binding protein 3 mitochondrial | A0A1D6MXZ7 | 26.02 | 4.80 | 19 | 262 | | | YGQVIEAR; LFVGGISYQTDDHSLR; GFGFVTYTSSEEASAAITALDGK; LFVGGISYQTDDHSLRDEFAK |
| 71 | Glycine-rich protein2 | AQK39923.1 | Glycine-rich protein2 | A0A1D6IV87 | 28.12 | 4.64 | 25 | 292 | | | YGQVIEAK; LFVGGLSYGTDDHSLR; LFVGGLSYGTDDHSLRDEFAK; GFGFITYTSSEEASAAITAMDGK; NDEIMDDLFKDDEPDSYANK |
| 72 | Glycine-rich RNA-binding protein 2 | ACG24633.1 | Glycine-rich RNA-binding protein 2 | B6SIF0 | 15.76 | 6.58 | 35 | 223 | | | EAFSSFGEVTEAR; GFGFVNYSDSDAAK; EAISAMDGKEIDGR; LFIGGLDWGVDDVK |
| 73 | Class I heat shock protein 3 | NP_001130454.1 | 17.4 kDa class I heat shock protein | B4F976 | 17.87 | 6.86 | 28 | 194 | | | TSSETAAFAGAR; ASMENGVLTVTVP; VEVEDGNVLQISGER; EEVKVEVEDGNVLQISGER |
| 74 | Cold shock protein 2 | AQK60690.1 | Cold shock protein 2 | C0PLI2 | 23.90 | 5.95 | 8 | 88 | | | GFGFISPEDGSEDLFVHQSSIK |
| 75 | Stress-inducible membrane pore protein | ONM31664.1 | Stress-inducible membrane pore protein | A0A1D6MRR5 | 17.68 | 6.41 | 35 | 473 | | | TLKDELTSMDR; NSAIAGAIAGAAVALTGDAGGHSDK; EAYVVTVEGLSGDSSGLDADGGK; EAYVVTVEGLSGDSSGLDADGGKR |
| 76 | Bowman-Birk type trypsin inhibitor | ONM30326.1 | Bowman-Birk type trypsin inhibitor | A0A1D6MM79 | 26.89 | 5.45 | 11 | 168 | | | YRGDPGPR; ECVETEDSR; TYPLTCSCFDR |
| 77 | Bowman-Birk type trypsin inhibitor | ONM30326.1 | Bowman-Birk type trypsin inhibitor | A0A1D6MM79 | 26.89 | 5.45 | 11 | 141 | | | YRGDPGPR; ECVETEDSR; TYPLTCSCFDR |
| 78 | Glycine-rich RNA-binding protein 3 mitochondrial | ONM33600.1 | Glycine-rich RNA-binding protein 3 mitochondrial | A0A1D6MXZ7 | 26.02 | 4.80 | 19 | 258 | | | YGQVIEAR; LFVGGISYQTDDHSLR; GFGFVTYTSSEEASAAITALDGK; LFVGGISYQTDDHSLRDEFAK |
| 79 | Oil body-associated protein 1A | NP_001132265.1 | Oil body-associated protein 1A | B4FFZ9 | 26.55 | 5.87 | 35 | 345 | | | TIHFWQVDR; TIHFWQVDR; EVDLPASTTAGAGR; QCLIFDGPGAGAR; GGVLFMPGVPGVVER; GGVLFMPGVPGVVER; GDALPLGLPQIMMVLTR; QVEAHHFCAHLNEDVR |
| 80 | Embryonic protein DC-8 precursor | NP_001146861.1 | Embryonic protein DC-8 | B6SGN7 | 32.37 | 8.39 | 19 | 165 | | | EAGHEAADR; VADQASGMAER; GVAEDAADTASR; VAETAQAIGDMAK; VVPDAEDVDAAVK |
| 81 | Hsp70-Hsp90 organizing protein 3 | AQK73114.1 | Hsp70-Hsp90 organizing protein 3 | A0A1D6HEP0 | 77.07 | 7.94 | 18 | 419 | | | NQELLDGVR; GEISQDELQER; DFEAAIQHYTK; DFDIAIETYQK; QVLNDFQENPR; ELEQQEYYDPK; NPSSISMYLSDPR; AYLNEPDFMQMLR; ALEHDDEDISYLTNR; EVEPEPEPEPMDFTDEEKER |
| 82 | 16.9 kDa class I heat shock protein 1 | NP_001146967.1 | 16.9 kDa class I heat shock protein 1 | B6SIX0 | 17.05 | 6.77 | 26 | 302 | | | FRLPENAK; AALENGVLTVTVPK; VEVEDGNVLLISGQR; EEVKVEVEDGNVLLISGQR |
| 83 | 16.9 kDa class I heat shock protein 1 | NP_001146967.1 | 16.9 kDa class I heat shock protein 1 | B6SIX0 | 17.05 | 6.77 | 24 | 190 | | | FRLPENAK; AALENGVLTVTVPK; VEVEDGNVLLISGQR |
| 84 | Bowman-Birk type trypsin inhibitor | ONM30326.1 | Bowman-Birk type trypsin inhibitor | A0A1D6MM79 | 26.89 | 5.45 | 7 | 104 | | | YRGDPGPR; TYPLTCSCFDR |
| 85 | Heat shock protein17.2 | ONM30195.1 | Heat shock protein17.2 | A0A1D6MLV9 | 13.04 | 6.62 | 35 | 314 | | | FRLPENAK; AGLENGVLTVTVPK; VEVEDGNVLVISGQR; EEVKVEVEDGNVLVISGQR |
| 86 | Embryonic protein DC-8 | ONL94071.1 | Embryonic protein DC-8 | A0A1D6JQ00 | 37.09 | 6.46 | 20 | 492 | | | LEEVKER; DAAWETVEAAK; HHADVDEEETAR; AQETLSQTADAAAEK; EAAEAASESGAEAHER; TKEAAEAASESGAEAHER |
| 87 | 1-Cys peroxiredoxin PER1 | A2SZW8.1 | 1-Cys peroxiredoxin PER1 | A2SZW8 | 25.06 | 6.31 | 48 | 656 | | | VTFPILADPAR; LSFLYPATTGR; LLGISCDDVESHR; QLNMVDPDEKDAAGR; MFPQGFETADLPSKK; PGLTIGDTVPNLELDSTHGK; VATPANWKPGECAVIAPGVSDEEAR; VATPANWKPGECAVIAPGVSDEEAR |
| 88 | Xyloglucan endotransglucosylase/hydrolase protein 32 precursor | NP_001148432.1 | Xyloglucan endotransglucosylase/hydrolase | B6T2W7 | 35.35 | 5.88 | 26 | 220 | | | NGYFGASVR; TLWGAQHQTLSPDGK; TLWGAQHQTLSPDGK; NSMVYYYCLDSTR; VGGCAADASADCQPVPASPAAPGTAAISAQQEAAMR |
| 89 | 60S acidic ribosomal protein P3 | NP_001105389.2 | 60S acidic ribosomal protein P3 | B4G1B3 | 12.18 | 4.45 | 29 | 264 | | | GVYTFVCR; NNGGEWTAK; QHSGEIEASAATPYELQR |
