# Supplementary material for: 2-DE-based proteomic analysis of protein changes associated with etiolated mesocotyl growth in Zea mays
Source: BMC Genomics. 2019 Oct 22;20:758. doi: 10.1186/s12864-019-6109-z (PMC6805590; doi:10.1186/s12864-019-6109-z)
Supplement: Supplementary file 3 — Additional file 3: Table S3. Primers used in RT-qPCR. [file 12864_2019_6109_MOESM3_ESM.docx]

| **Protein** | **Sense primer** | **Anti-sense primer** |
| --- | --- | --- |
| Vacuolar proton pump 3 (spot 5) | 5' GATAAGTTCTGCCCATTCT 3' | 5' GTGCCTCATCTTCTAGGTT 3' |
| Golgi associated protein-like protein (spots 13, 47) | 5' TCCCTGACTATGATGCTCC 3' | 5' CCAGAAGATGCCCTTGTATT 3' |
| Xyloglucan endotransglycosylase (spot 22) | 5' AGAAGTTCACGACGGAGGG 3' | 5' AAGCACCGCACGAAGAGG 3' |
| UDP-arabinopyranose mutase 3 (spot 50) | 5' TTCGGTCTCATGGGTGAT 3' | 5' TCTGGACGGTGTCGCAAT 3' |
| Cysteine synthase (spot 44) | 5' TCCTTACTGACCCACTCTT 3' | 5' ACCAACAACCCTTCTTTC 3' |
| Cysteine proteinase 2 (spot 52) | 5' CGGAGACGAAAGACTGGA 3' | 5' CAACACTAACTGGGCGAAC 3' |
| Ubiquitin (reference) | 5' TAAGCTGCCGATGTGCCTGCG3' | 5' CTGAAAGACAGAACATAATGAGCACAG 3' |

**Table S3** Primers used in RT-qPCR
