# Supplementary figures and images for: 2-DE-based proteomic analysis of protein changes associated with etiolated mesocotyl growth in Zea mays
Source: BMC Genomics. 2019 Oct 22;20:758. doi: 10.1186/s12864-019-6109-z (PMC6805590; doi:10.1186/s12864-019-6109-z)

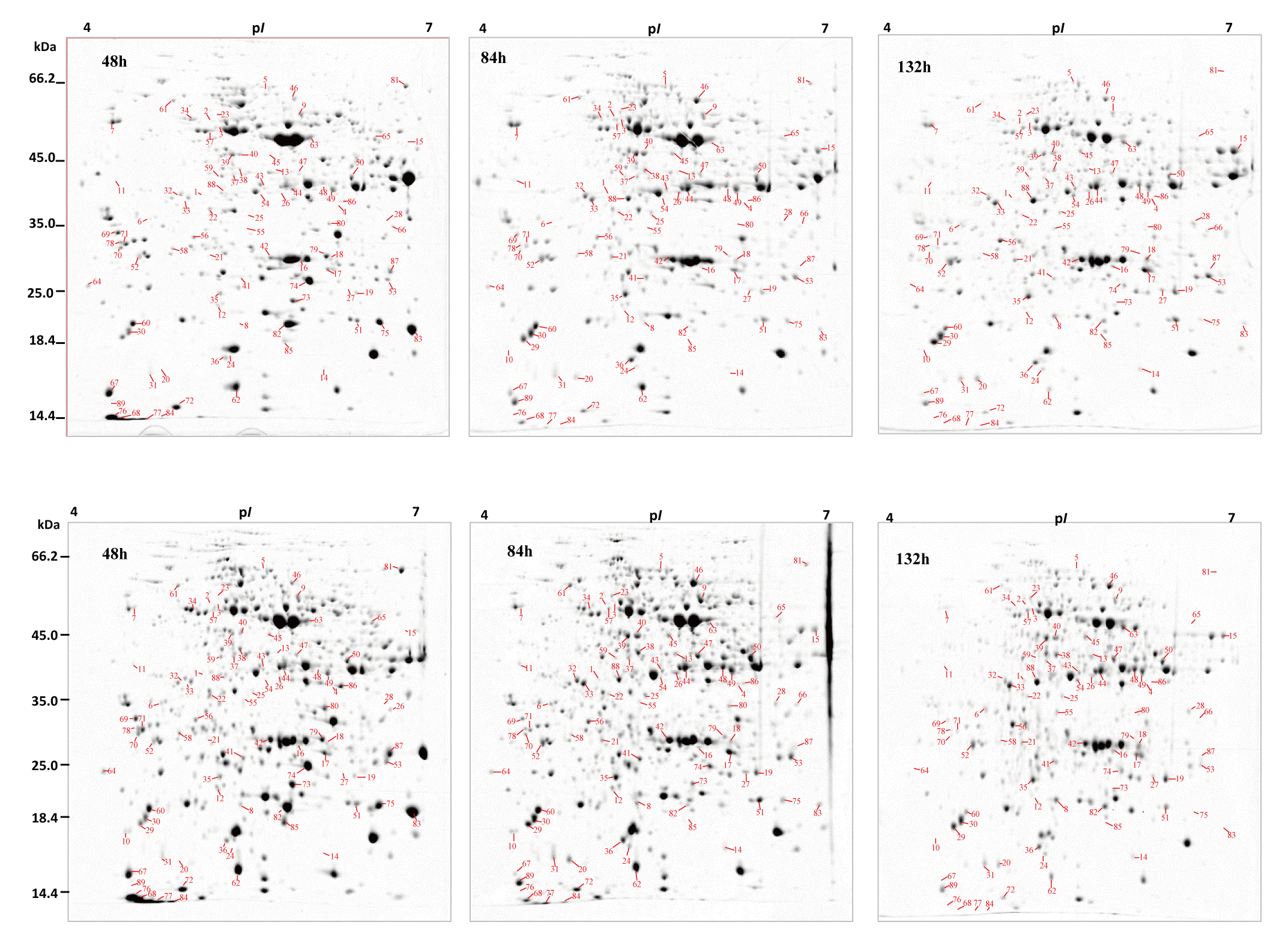

Supplement: Supplementary file 4 — Additional file 4: Figure S1. The 2-DE profiles of maize mesocotyl at 48 h, 84 h and 132 h. Maize mesocotyl proteins (600 μg) were resolved by IEF using 11 cm pH 4–7 IPG dry strips. Secondary SDS-PAGE was carried out on a 12.5% resolving gel. The proteins were visualized using CBB R350 staining. Spots of relatively abundant proteins in the maize mesocotyl at 48 h, 84 h and 132 h with at least a two-fold change in abundance are indicated with a red line. [file 12864_2019_6109_MOESM4_ESM.tif]

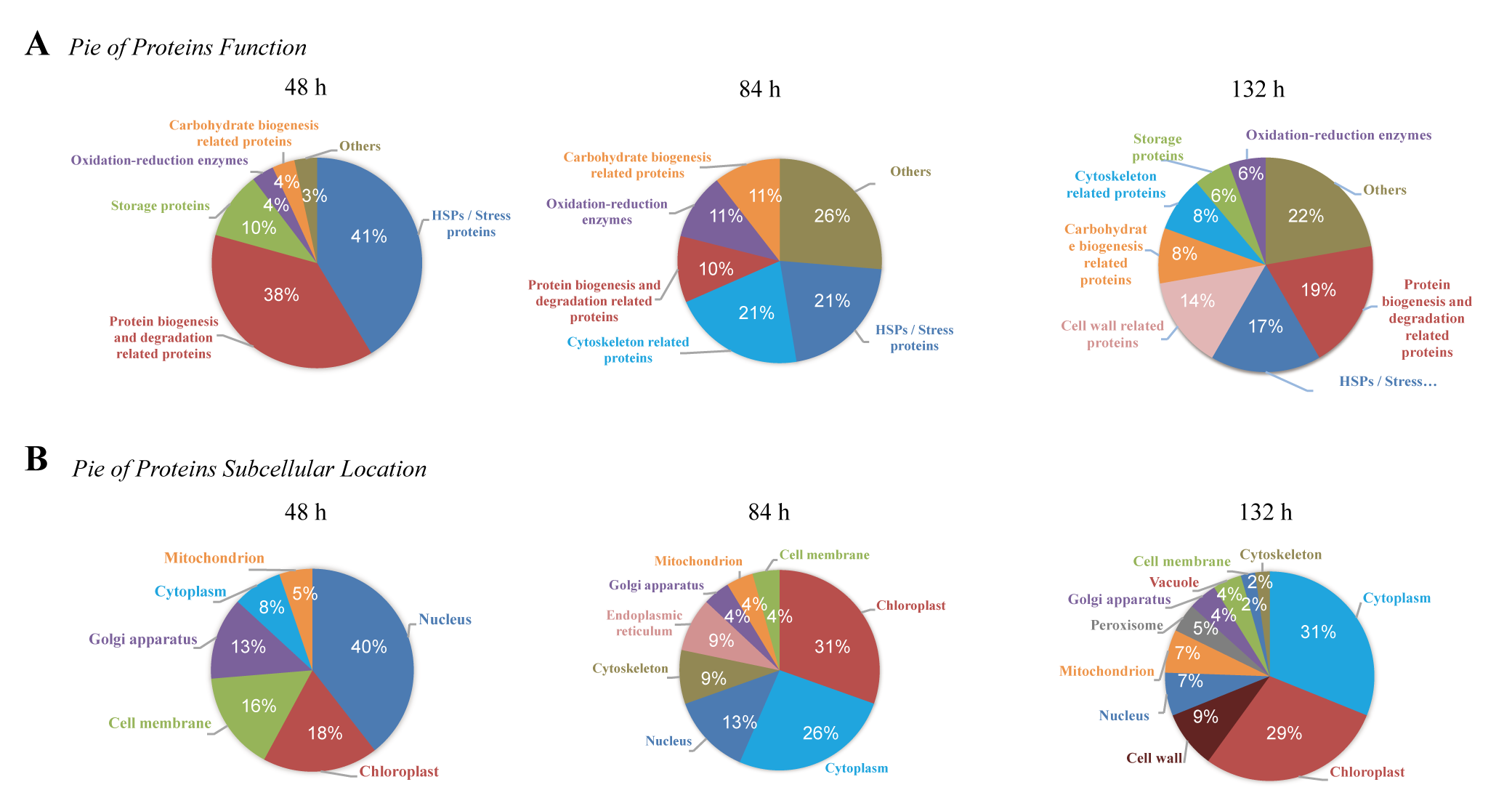

Supplement: Supplementary file 5 — Additional file 5: Figure S2. Pie charts of the DAPs identified from the maize mesocotyl. A, Classification of the DAPs based on molecular function. B, Classification of the DAPs based on subcellular localization. [file 12864_2019_6109_MOESM5_ESM.tif]
